# NprR-NprX Quorum-Sensing System Regulates the Algicidal Activity of *Bacillus* sp. Strain S51107 against Bloom-Forming Cyanobacterium *Microcystis aeruginosa*

**DOI:** 10.3389/fmicb.2017.01968

**Published:** 2017-10-11

**Authors:** Lishuang Wu, Xingliang Guo, Xianglong Liu, Hong Yang

**Affiliations:** State Key Laboratory of Microbial Metabolism, School of Life Sciences and Biotechnology, Shanghai Jiao Tong University, Shanghai, China

**Keywords:** harmful cyanobacterial blooms, *Microcystis aeruginosa*, algicidal bacteria, Gram-positive bacteria, quorum sensing, algicidal compound

## Abstract

Harmful cyanobacterial blooms have severely impaired freshwater quality and threatened human health worldwide. Here, a Gram-positive bacterium, *Bacillus* sp. strain S51107, which exhibits strong algicidal activity against *Microcystis aeruginosa*, was isolated from Lake Taihu. We found that the algicidal activity of strain S51107 was regulated primarily by NprR-NprX quorum sensing (QS), in which the mature form of the signaling peptide NprX was identified as the SKPDIVG heptapeptide. Disruption of the *nprR-nprX* cassette markedly decreased the algicidal activity, and complemented strains showed significantly recovered algicidal activity. Strain S51107 produced low-molecular-weight algicidal compounds [indole-3-carboxaldehyde and cyclo(Pro-Phe)] and high-molecular-weight algicidal substance(s) (>3 kDa). Moreover, the production of high-molecular-weight algicidal substance(s) was regulated by NprR-NprX QS, but the production of low-molecular-weight algicidal compounds was not. High-molecular-weight algicidal substance(s) played a more important role than low-molecular-weight algicidal compounds in the algicidal activity of strain S51107. The results of this study could increase our knowledge about algicidal characteristics of a potential algicidal bacterium, *Bacillus* sp. strain S51107, and provide the first evidence that the algicidal activity of Gram-positive algicidal bacteria is regulated by QS, which will greatly enhance our understanding of the interactions between algae and indigenous algicidal bacteria, thereby providing aid in the design and optimization of strategies to control harmful algae blooms.

## Introduction

Harmful cyanobacterial blooms (CyanoHABs), which produce toxins, drive hypoxia, disrupt the food web and generate other noxious substances, have increased globally in freshwater systems (Paerl and Otten, 2016). Hypereutrophic Lake Taihu, China’s third-largest freshwater lake, has experienced persistent harmful cyanobacterial blooms dominated by *Microcystis* spp., which account for more than 90% of the total algal biovolume during the summer; these blooms threaten human health and destroy aquatic ecosystems (Qin et al., 2015; Ma et al., 2016). Consequently, control of toxic cyanobacterial expansion is a pressing need. Biological methods have attracted considerable attention in recent years as a potentially effective strategy to control CyanoHABs because these methods may be more targeted and be native to aquatic environments (Jung et al., 2008; Sengco, 2009). Among all the organisms, including hydrophytes, zooplankton, viruses, and bacteria, algicidal bacteria play an important role in lysing algal cells in aquatic ecosystems (Kang et al., 2005; Li Y. et al., 2015; Zhang et al., 2016).

Previous studies have reported that some bacteria must reach a threshold density before they show algicidal activity against harmful algae, such as Gram-negative *Cytophaga* strain 41-DBG2 (Mayali and Doucette, 2002), *Pseudomonas putida* HYK0203-SK02 (Kang et al., 2005), *Shewanella* sp. Lzh-2 (Li et al., 2014) and *Stenotrophomonas* F6 (Lin et al., 2016), and Gram-positive *Rhodococcus* sp. p52 (Wang et al., 2013) and *Bacillus* sp. Lzh-5 (Li Z. et al., 2015). Skerratt et al. (2002) have reported that algicidal *Bacillus cereus* ACEM 32 produces algicidal exudates only in the stationary phase, thus indicating that the lytic effects of these algicidal bacteria are cell-density-dependent. Therefore, research on the cell-density-dependent quorum-sensing system in algicidal bacteria is crucial for the effective control of CyanoHABs.

Quorum sensing (QS) is a cell-cell communication process in which bacteria produce, release, detect, and respond to extracellular signaling molecules in a cell-density-dependent manner (Waters and Bassler, 2005; Hoover et al., 2015). Gram-negative bacteria primarily produce acyl homoserine lactones (AHLs) as signaling molecules (Ng and Bassler, 2009), whereas Gram-positive bacteria primarily use modified oligopeptides. In the algicidal Gram-negative γ-*proteobacterium* strain MS-02-063, production of the algicidal pigment PG-L-1 is controlled by homoserine lactone quorum sensing (Nakashima et al., 2006). Recently, Guo et al. (2016) have confirmed that the production of two algicidal compounds by *Aeromonas* sp. strain GLY-2107 is regulated by *N*-butyryl-homoserine lactone (C4-HSL)-mediated QS. Harvey et al. (2016) have reported that an alkylquinolone-mediated quorum-sensing precursor, 2-heptyl-4-quinolone (HHQ), which is released by *Pseudoalteromonas piscicida*, induces the mortality of *Emiliania huxleyi* (Harvey et al., 2016). However, only QS systems of Gram-negative bacteria have been implicated in algicidal activity. It is not known whether quorum signaling plays a role in the regulation of the algicidal activity of Gram-positive bacteria, an important group of algicidal bacteria (Skerratt et al., 2002; Salomon et al., 2003; Kim et al., 2008; Li Z. et al., 2015). Therefore, understanding whether the QS system of Gram-positive algicidal bacteria activates and deactivates their algicidal ability could aid in the control of harmful cyanobacterial blooms.

Gram-positive bacteria generally use oligopeptides as signaling molecules in QS-controlled gene expression systems. Secreted peptides are detected at the bacterial surface by two-component regulatory systems or are reimported by an oligopeptide transport system and subsequently interact with cognate intracellular regulators (Monnet and Gardan, 2015). The latter group of quorum-sensing regulators form the RRNPP (Rgg/Rap/NprR/PlcR/PrgX) family and a new Tprs (Transcription factor regulated by a Phr peptide) family, which have been investigated in bacilli, enterococci, and streptococci (Kozlowicz et al., 2006a; Bouillaut et al., 2008; Gallego del Sol and Marina, 2013; Hoover et al., 2015; Parashar et al., 2015). Members of this family regulate several important processes, such as virulence gene expression, sporulation, biofilm formation, conjugation, development of competence, and production of extracellular proteins (Pottathil and Lazazzera, 2003; Kozlowicz et al., 2006b; Mashburn-Warren et al., 2010; Perchat et al., 2011; Grenha et al., 2013).

In this study, a strain of Gram-positive algicidal bacteria, *Bacillus* sp. strain S51107, was isolated from Lake Taihu. We aim to investigate the effect of the quorum-sensing system on the algicidal activity of the strain S51107, the production of algicidal compounds of the strain, and the algicidal characteristics of the strain against an indigenous *Microcystis aeruginosa* from Taihu, which produced the toxin microcystin-LR.

## Materials and Methods

### Algal and Bacterial Strains and Growth Conditions

*Microcystis aeruginosa* 9110 (CGMCC 9118) (microcystin-LR produced by *M. aeruginosa* 9110 was detected using UPLC-MS/MS method described by Greer et al. (2016)), *Synechococcus* sp. BN60 (CGMCC 9117), *Oscillatoria* sp. BN35, and *Chlamydomonas* sp. BS3 were isolated from Meiliang Bay in Lake Taihu. *Microcystis viridis* FACHB-979, *Chroococcus* sp. FACHB-191, and *Microcystis aeruginosa* PCC7806 were obtained from the Freshwater Algae Culture Collection of the Institute of Hydrobiology (FACHB; Chinese Academy of Sciences, China). All the cyanobacterial and algal strains were incubated in BG11 medium (Stanier et al., 1971) at 25°C under 40 μmol photons/m^2^/sec and a 12h:12h (light:dark) cycle (Tian et al., 2012).

The bacterial strains used in this study are listed in Supplementary Table S1. *Escherichia coli* DH5α was used for cloning and sequencing manipulations. The plasmid used for the electroporation of *Bacillus* sp. in this study was prepared from *E. coli* SCS110. *E. coli* strains were grown at 37°C with shaking at 180 rpm in Luria broth (LB). *Bacillus* sp. S51107 and *B. thuringiensis* 407 Cry^-^ (*nprA′Z* ΔRX [pHT304-R]) were grown in LB or beef extract peptone (BEP; Peptone 10 g, NaCl 5 g, and yeast extract 3 g per liter, pH 7.5) at 28°C and 200 rpm unless indicated otherwise. The antibiotic concentrations for bacterial selection were as follows: ampicillin, 100 μg/ml and streptomycin, 25 μg/ml (for *E. coli*); erythromycin, 5 μg/ml (for *Bacillus*); tetracycline, 10 μg/ml (Sangon Biotech, China).

### Chemicals

The Pfu PCR MasterMix was obtained from TIANGEN Biotech (Beijing, China). The restriction enzymes and T4 DNA ligase were obtained from Takara (Dalian, China). The oligonucleotide primers used for PCR amplification were synthesized by Liuhe BGI Tech Co. Ltd. (Shanghai, China). DNA sequences were determined by Liuhe BGI Tech Co. Ltd. Standard indole-3-carboxaldehyde and other chemicals were obtained from Sigma–Aldrich (United States) unless otherwise specified. Cyclo(Pro-Phe) (3-benzyl-hexahydro-pyrrolo[1,2-a]pyrazine-1,4-dione) and NprX signaling peptide were synthesized by GL Biochem Ltd. (Shanghai, China).

### Isolation, Screening and Identification of Algicidal Bacteria

The algicidal bacteria were isolated from the hypertrophic Meiliang Bay in Lake Taihu (31°24′N, 120°13′E) in March 2014. Water samples were collected into sterile bottles using a Ruttner Standard Water Sampler, and transported to laboratory in a cooler. Ten-milliliter water sample was added into 90-ml *M. aeruginosa* 9110 cultures in exponential growth phase, and incubated under cyanobacterial growth conditions. An equal volume of sterile distilled water was used instead of water sample as the negative control. When the cell density of *M. aeruginosa* 9110 declined to below 10% of that in negative control, 1-ml aliquots of the co-culture were serially diluted with sterile water and spread onto BEP agar plates, and the plates were incubated at 30°C for 24 h. Individual colonies with distinct morphology were streaked onto BEP plates for further purification. After that, each colony was preserved at -70°C in BEP liquid medium containing 25% glycerol.

Each isolated bacterial strain was inoculated into a sterile test tube containing 3 ml of BEP liquid medium and cultivated at 28°C and 220 rpm for 24 h. Then, 200 μl of above bacterial culture was added into a shake flask containing 9.8 ml of exponential-phase *M. aeruginosa* 9110. The negative control was added with an equal volume of sterile BEP medium instead of bacterial culture. After 6 days of co-culture, the algicidal activity was evaluated.

### Determination of Algicidal Activity

The cell densities of *M. aeruginosa* were counted with a hemocytometer under a light microscope (Olympus, Japan). The chlorophyll-*a* concentrations, as the equivalent cell densities of other cyanobacteria, were determined spectrophotometrically by using the acetone method (Welschmeyer, 1994). The algicidal activity (*A*, %) was calculated by using the following equation (Li Z. et al., 2015): *A* = (1 - *D_t-treatment_*/*D_t-control_*) × 100, where *D_t-treatment_* and *D_t-control_* are the cell densities of cyanobacteria with the treatment and control, respectively, and *t* is the incubation time.

### Determination of the Algicidal Mode and Range of Strain S51107

The algicidal mode of strain S51107 against *M. aeruginosa* 9110 was determined according to the procedures described previously (Guo et al., 2016) with some modifications. The culture of strain S51107 in stationary phase, which was cultivated in BEP medium at 30°C and 200 rpm, was centrifuged at 3,000 × *g* for 10 min. The supernatant was passed through a 0.22-μm cellulose acetate membrane filter to obtain cell-free filtrate. The heat-treated cell-free filtrate was prepared by autoclaving at 121°C for 20 min. The bacterial cells were collected by centrifugation (3000 × *g*, 10 min), washed twice with sterile BG11 medium, and re-suspended in an equal volume of BG11 medium. An aliquot (200 μl) of bacterial cultures, cell-free filtrates, heat-treated cell-free filtrates, and re-suspended bacterial cells was inoculated into 9.8 ml log-phase *M. aeruginosa* 9110 culture and cultivated under algal growth conditions. As a control, 200 μl of BEP medium was inoculated into a 9.8-ml *M. aeruginosa* 9110 culture. The algicidal rates were calculated on day 6 of inoculation.

The algicidal range assay of strain S51107 was performed according to a method previously described by Guo et al. (2016). Briefly, Strain S51107 was grown in BEP medium to late exponential phase (approximately OD_600nm_ of 4.5 in a 1-cm cell, using a spectrophotometer) at 30°C and 200 rpm; the harvested bacterial cells were washed twice with BEP medium and re-suspended in BEP medium, thus resulting in a 100-fold dilution of the bacterial suspension; then, 200 μl of bacterial suspensions were added to 9.8-ml log-phase tested algal cultures and cultivated under algal growth conditions. At the same time, 200-μl BEP medium was added to the tested algal cultures as the corresponding control.

### Transmission Electron Microscopy

Twenty milliliters of strain S51107 and *M. aeruginosa* 9110 co-cultures was collected at 3,000 × *g* and 25°C for 5 min and fixed in 0.1 M phosphate buffer (PB, pH 7.4) containing 2.5% glutaraldehyde (v/v) for 6 h before being washed three times with 0.1 M PB buffer. The samples were post-fixed in 2% osmium tetroxide in 0.1 M PB buffer for 2 h; gently rinsed three times with PB buffer; and dehydrated with 50% ethanol (15 min), 70% ethanol (15 min), 90% ethanol (15 min), 90% ethanol/90% acetone (1:1, v/v, 20 min), and 90% acetone (20 min) and three times with 100% acetone (20 min) (Lei et al., 2015). The samples were embedded in araldite resin. Sections were cut on an ultramicrotome and stained in 3% acetic acid uranium-citric acid. TEM images were captured using a 120 kV biology transmission electron microscope (FEI, Tecnai G2 Spirit BioTwin).

### Cloning of an *nprR-nprX* Quorum Sensing Genes in *Bacillus* sp. Strain S51107, and Identification of NprX Peptide from *Bacillus* sp. Strain S51107

To obtain the *nprR* and *nprX* genes from *Bacillus* sp. strain S51107, primers Npr1-F and Npr2-R (Supplementary Table S2) were designed according to the genomic sequences of *B. thuringiensis* strain 407 (GenBank accession number of NprR regulator and NprX signaling peptide genes: NC_018877.1: 604770-607345). The *nprR* and *nprX* genes were amplified by PCR from the genome of strain S51107 using the primers Npr1-F and Npr2-R. The genes were ligated to the pMD18-T vector (Takara, Dalian, China) and then sequenced.

The reporter strain *B. thuringiensis* 407 Cry^-^ (*nprA′Z* ΔRX [pHT304-R]) lacks the ability to express the NprX peptide (Perchat et al., 2011). This strain contains the *nprA* promoter fusion to *lacZ* gene, and *nprA* belongs to the NprR regulon. Thus, a strain that secretes an NprX peptide specifically able to activate the NprR regulator of the reporter strain is able to restore the β-galactosidase production of the reporter strain, and this complemented reporter strain should appear blue on an agar plate supplemented with X-Gal. The reporter strain was streaked into an “L” shape on an BEP agar (1.5%, wt/vol) plate supplemented with X-Gal (5-bromo-4-chloro-3 indolyl-β-D-galactopyranoside; 100 μg/ml; Sangon Biotech, China), and the algicidal strains tested were streaked in parallel on one side of the reporter strain. After 24 h of inoculation at 30°C, restoration of β-galactosidase by the reporter strain was observed.

Strain S51107 was grown in BEP medium at 30°C and 200 rpm, and the supernatant was harvested by centrifugation (20 min, 3000 × *g*) 4 h after the onset of the stationary phase and passed through a 0.22-μm membrane filter. The NprX peptides from filtrate of strain S51107 and BEP medium (control) were roughly extracted by solid-phase extraction on Sep-Pak Vac/3cc C18 cartridges (Waters, United States) using a 50% methanol solution for the elution step. The peptides were then concentrated by lyophilization. The dried fractions were re-suspended in 20% methanol in water and tested for the presence of NprX on BEP agar plates with X-Gal and reporter strain. The peptides from strain S51107 were identified by LC-MS/MS with a Finigan Surveyor liquid chromatograph (LC) (Thermo Fisher, United States) system coupled with a Linear Ion Trap Quadrupole (LTQ) XL Mass Spectrometer (Thermo Fisher, United States) fitted with an electrospray ionization (ESI) source (see Supplementary Materials and Methods for details).

### Construction of *Bacillus* sp. S51107 Recombinant Strains

The *nprR-nprX* genes were inactivated by a deletion that eliminates the coding sequence without insertion of foreign DNA by homologous recombination by using the thermosensitive allele exchange plasmid pMAD-RX, which was constructed as the follows. The 5′ upstream and 3′ downstream regions of the *nprR-nprX* locus were amplified by using the NPRX1-F/NPRX2-R and NPRX3-F/NPRX4-R primer pairs. The two fragments were ligated by overlap extension PCR and inserted into pMAD between BamHI and NcoI. The pMAD-RX plasmid was introduced by electroporation into strain S51107, as described by Liu et al. (2014), and transformants were selected at 30°C on LB plates containing erythromycin (5 μg/ml) and X-Gal (50 μg/ml). The *nprR-nprX* mutant was obtained as the procedures described by Arnaud et al. (2004). Briefly, one blue clone was inoculated in LB liquid medium containing erythromycin and X-Gal and cultivated at 40°C. Serial dilutions were plated on LB plates (containing erythromycin and X-Gal) and incubated at 40°C. Clones resulting from the integration of pMAD-RX plasmid into the *nprR-nprX* genes (a single crossover event via homologous sequences) were blue. Several blue colonies were cultivated at 30°C for 6 h, followed by cultivation at 40°C for 3 h; dilutions were spread on LB plates without antibiotics. The white colonies on X-Gal plates resulting from a double crossover events have undergone the loss of the plasmid vector. Several white colonies were tested for erythromycin sensitivity and confirmed the gene deletion by PCR amplification and sequencing with Npr1E-F and NprR-2 primers that flank the disrupted genes. Genetic complementation of the *nprR-nprX* mutant was performed by introducing the pHT304-R plasmid and pHT304-RX plasmid into the *nprR-nprX* mutant, respectively, to obtain the genetically complemented *Bacillus* sp. strain *nprR-nprX* [pHT304-R] mutant and *Bacillus* sp. strain *nprR-nprX* [pHT304-RX] mutant. The control strain (strain *nprR-nprX* [pHT304] mutant) was acquired by introducing the pHT304 plasmid into the *nprR-nprX* mutant. All the primers and plasmids were listed in Supplementary Tables S2 and S3, respectively.

### Investigation of the Role of the NprR-NprX QS System in the Algicidal Process of *Bacillus* sp. Strain S51107

The preparations of bacterial suspensions from the wild type strain S51107, *nprR-nprX* mutant, strain *nprR-nprX* [pHT304-R] mutant, strain *nprR-nprX* [pHT304-RX] mutant, and the corresponding control strain *nprR-nprX* [pHT304] mutant were performed by the method described by Guo et al. (2016) with some modifications. Each strain was cultured separately in BEP medium to late exponential phase at 30°C and 200 rpm. Then, each of the cell cultures was centrifuged at 3000 × *g* for 10 min at 25°C. The cell pellets were washed twice with sterile fresh BEP medium and re-suspended in sterile fresh BEP medium, thus resulting in a 100-fold dilution of the bacterial suspension. Two milliliters of each bacterial suspension was inoculated into 98 ml of *M. aeruginosa* 9110 (2 × 10^6^ cells/ml). The chemical complementation of the strain *nprR-nprX* [pHT304-R] mutant was conducted by exogenous addition of 5 μM synthetic peptide SKPDIVG, as described in a previous study on the *nprX*-deficient strain of *B. thuringiensis* (Perchat et al., 2011). A stock solution of the standard NprX peptide was dissolved in BG11 medium to 50 mM. An aliquot (10 μl) of fresh NprX stock solution with 2 ml bacterial suspension of *nprR-nprX* [pHT304-R] mutant was inoculated into 97.99 ml of *M. aeruginosa* 9110. The corresponding control sample had 2 ml BEP medium added instead of the bacterial suspension. All of the co-cultures were cultured under algal growth conditions. The cell densities of *M. aeruginosa* 9110, the algicidal bacterial strain S51107 and its derived mutants, and the concentrations of the algicidal compounds and dissolved organic carbon (DOC) were determined daily during the 6-day algicidal process.

The DOC was determined through the following procedure: the sampled culture was centrifuged at 3000 × *g* for 10 min at 25°C, and the supernatant was filtered through a 0.22-μm membrane filter before the filtrate was analyzed for DOC on a Multi 3100 N/C TOC analyzer (Analytik Jena, Germany).

### Isolation, Purification, and Identification and Dose Response Bioassays of Bacterial Algicidal Compounds; Quantification Analysis of Algicidal Compounds from Strain S51107 during the Lysing Process

The isolation, purification, identification and dose response bioassays of algicidal compounds, and quantification of algicidal compounds in the co-cultures were accomplished using the methods described in our previous work (Guo et al., 2016) with some modifications and detailed in Supplementary Materials and Methods.

### Algicidal Activity of Size-Fractionated Cell-Free Filtrates

Cultures of strain S51107, *nprR-nprX* mutant, *nprR-nprX* [pHT304-R] mutant, *nprR-nprX* [pHT304-R] mutant supplemented with 5 μM SKPDIVG peptide, *nprR-nprX* [pHT304-RX] mutant and *nprR-nprX* [pHT304] mutant in the stationary phase were centrifuged at 3,000 × *g* for 10 min, and the supernatants were filtered using 0.22-μm syringe filters. Fifty milliliters of cell-free supernatants were lyophilized and then fractionated with Amicon Ultra centrifugal filter units with a 3 kDa molecular weight cut off (Millipore, United States) as described in the manufacturer’s instructions. The low- and high-molecular-weight fractions were diluted to 1.5 ml with BG11 medium. The algicidal activities of both fractions were determined in 96-well plates by inoculation of 240 μl of fractionated filtrates with 60 μl of exponential phase *M. aeruginosa* 9110 (Paul and Pohnert, 2011).

### Data Analysis

Each value represents the mean ± SD based on three replicates, and the bars indicate the standard deviations (SD). Statistical comparisons between different groups were made by using one-way ANOVA (SPSS v 20.0) with Dunnett’s test. A probability level of *P* < 0.05 was considered statistically significant.

### Accession Number(s)

The 16S rRNA, *nprR*, and *nprX* gene sequences of strain S51107 have been submitted to GenBank database under accession numbers KT895988, KY412437, and KY412438, respectively. The S51107 strain has been deposited in the China General Microbiological Collection Center (CGMCC) under accession number CGMCC-12147.

## Results

### Isolation and Identification of Algicidal Bacteria

Eleven bacterial strains isolated from water samples collected from Meiliang Bay in Lake Taihu (in March 2014) showed strong algicidal activity against *M. aeruginosa* 9110. Strain S51107, which exhibited the strongest algicidal activity (*A*) (*A* = 92.5%, *t* = 6 days) among the Gram-positive bacteria, was chosen for further research.

16S rRNA gene sequence comparisons performed with the sequences available in the GenBank database, revealed that strain S51107 was most closely related to *B. cereus* ATCC 14579^T^, with 100% identity (GenBank accession number AE016877). Phase-contrast microscopy revealed rod-shaped and spore-forming bacteria with rounded ends, found singly or in short or long chains (Supplementary Figure S1). This morphology was in accordance with bacilli and phylogenetic analysis (Supplementary Figure S2). Therefore, the new strain was named *Bacillus* sp. strain S51107.

### Algicidal Range and Mode of *Bacillus* sp. Strain S51107

**FIGURE 1:**
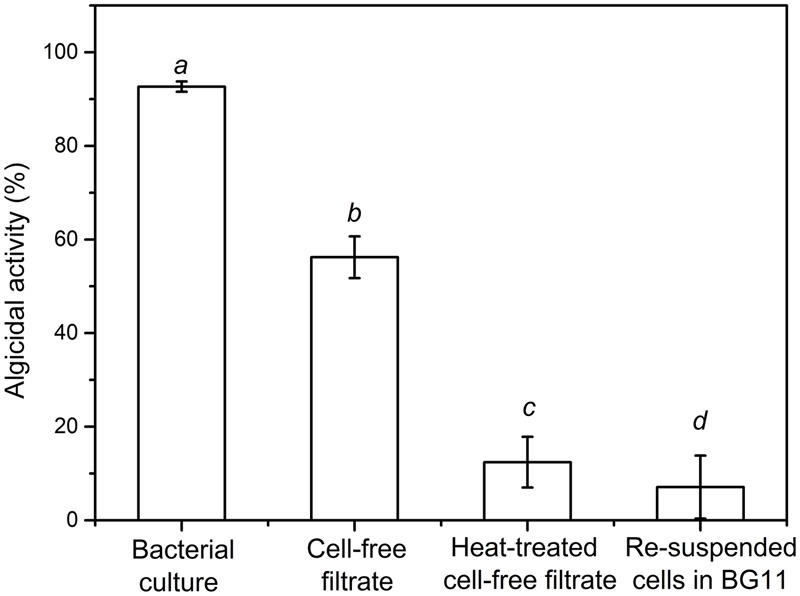
Algicidal activities of different treatments of *Bacillus* sp. strain S51107 cultures against *Microcystis aeruginosa* 9110 after 6 days of inoculation. The data are the averages of three independent experiments (error bars are the SD from mean values). Different letters indicate statistically significant differences (*P* < 0.05).

Strain S51107 displayed high algicidal effects against the dominant cyanobacterial species from Meiliang Bay of Lake Taihu and other cyanobacterial species tested (**Table 1**). As shown in **Figure 1**, strain S51107 cultures showed a little higher algicidal activity against *M. aeruginosa* 9110 (*A* = 92.5%, *t* = 6 days) compared with its cell-free filtrates (*A* = 58.3%, *t* = 6 days), while it showed a far higher algicidal activity than that of washed cells of strain S51107 (*A* = 7.1%, *t* = 6 days) (*P* < 0.01). Moreover, the algicidal activity of the cell-free filtrates was higher than that of heat-treated cell-free filtrates (*A* = 11.2%, *t* = 6 days) (*P* < 0.01). These results suggested that the algicidal activity of strain S51107 occurs primarily via secretion of extracellular algicidal substances, which might involve thermolabile compounds.

**Table 1:** Algicidal effect of the isolate strain S51107 against algal or cyanobacterial strains.

Strains	Cell density (×10^6^ cells/ml) or chl *a* concentration (mg/l)^∗^		Algicidal activity (*A*, %)
	Control	Treatment	
*Microcystis aeruginosa* 9110	2.05 ± 0.09	0.15 ± 0.05	92.51 ± 2.79
*Synechococcus* sp. BN60	8.05 ± 0.25	1.43 ± 0.27	82.25 ± 3.06
*Chlamydomonas* sp. BS3	5.99 ± 0.18	2.69 ± 0.34	55.06 ± 6.63
*Oscillatoria* sp. BN35	7.37 ± 0.07	1.16 ± 0.11	84.22 ± 1.35
*Chroococcus* sp. FACHB-191	7.38 ± 0.20	1.74 ± 0.26	76.40 ± 4.10
*Microcystis viridis* FACHB-979	6.43 ± 0.20	1.08 ± 0.20	83.21 ± 3.20
*Microcystis aeruginosa* PCC7806	2.31 ± 0.18	0.19 ± 0.02	91.65 ± 1.00

*^∗^The cell densities of *Microcystis aeruginosa* were counted using a hemocytometer and other cyanobacteria were calculated using chlorophyll *a* concentration. The data are the mean ± SD from triplicate biological replicates.*

### Effects of *Bacillus* sp. Strain S51107 on the Ultrastructure of *M. aeruginosa*

**FIGURE 2:**
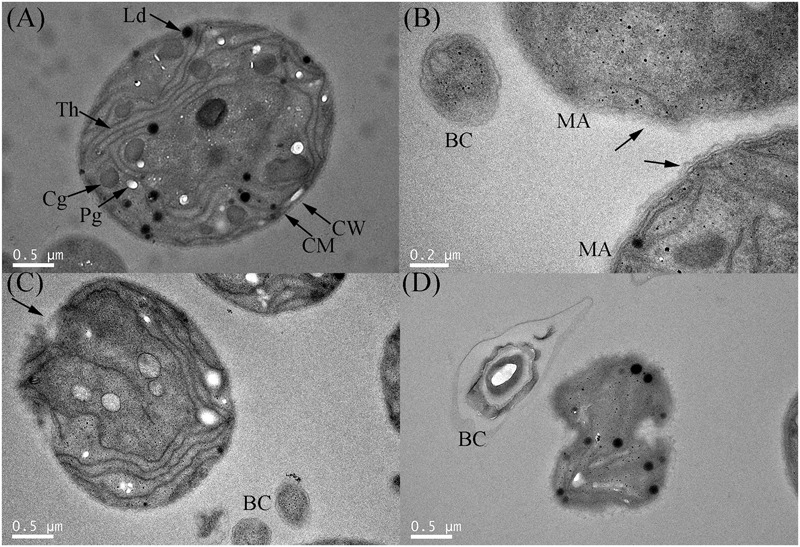
TEM observations of the degradation process of *M. aeruginosa* treated with *Bacillus* sp. strain S51107. **(A)** Normal cell; **(B)** damaged cell wall; **(C)** ruptured cell wall and membrane; **(D)** distorted and broken cell. CW, cell wall; CM, cell membrane; Th, thylakoids; Ld, lipid droplets; Cg, cyanophycin granules; Pg, polyphosphate granules; BC, bacterial cells; MA, *M. aeruginosa*. The arrows in **(B)** show damaged (up) and intact (down) cell walls. The arrows in **(C)** indicate the partly ruptured *M. aeruginosa* cell.

Transmission electron microscopy (TEM) was applied to observe the ultrastructural changes of *M. aeruginosa* after exposure to strain S51107 (**Figure 2**). As shown in **Figure 2A**, the control normal algal cells revealed intact cell wall and cell membrane surfaces, and typical and regular internal structures were present, including thylakoids, lipid droplets, cyanophycin granules, and polyphosphate granules. Under the algicidal effect of strain S51107, the surface structure of *M. aeruginosa* cells was missing, and the cell wall and membrane were deformed and partly ruptured (**Figures 2B,C**). Eventually, the cell morphology was highly distorted, and the cellular structure disappeared (**Figure 2D**).

### Identification of the NprR-NprX Quorum Sensing Genes in *Bacillus* sp. Strain S51107

The sequence analysis of *nprR-nprX* genes showed that the *nprR* gene was located upstream from the *nprX* gene in *Bacillus* sp. strain S51107 (Supplementary Figure S3). We identified the NprR regulating protein (423 amino acids) and NprX signaling peptide (43 amino acids). The NprR protein amino acid sequence of strain S51107 was highly similar to that of *B. thuringiensis* Bt407 (99%, GenBank accession number NC_018877.1). The presumed mature form of the NprX heptapeptide (SKPDIVG) of strain S51107 was the same as that of *B. thuringiensis* Bt407. This heptapeptide sequence activates expression of the NprR regulon in the reporter strain (Perchat et al., 2011; Rocha et al., 2012).

### Identification of NprX Peptide Produced by *Bacillus* sp. Strain S51107

As shown in Supplementary Figure S4, the reporter strain *B. thuringiensis* 407 Cry^-^ (*nprA*′*Z* ΔRX [pHT304-R]) cells grown close to the tested strain *Bacillus* sp. S51107 displayed a blue coloration, showing that *lacZ* expression was restored in these cells of reporter strain. It suggested that *Bacillus* sp. S51107 secreted active NprX signaling peptide.

**FIGURE 3:**
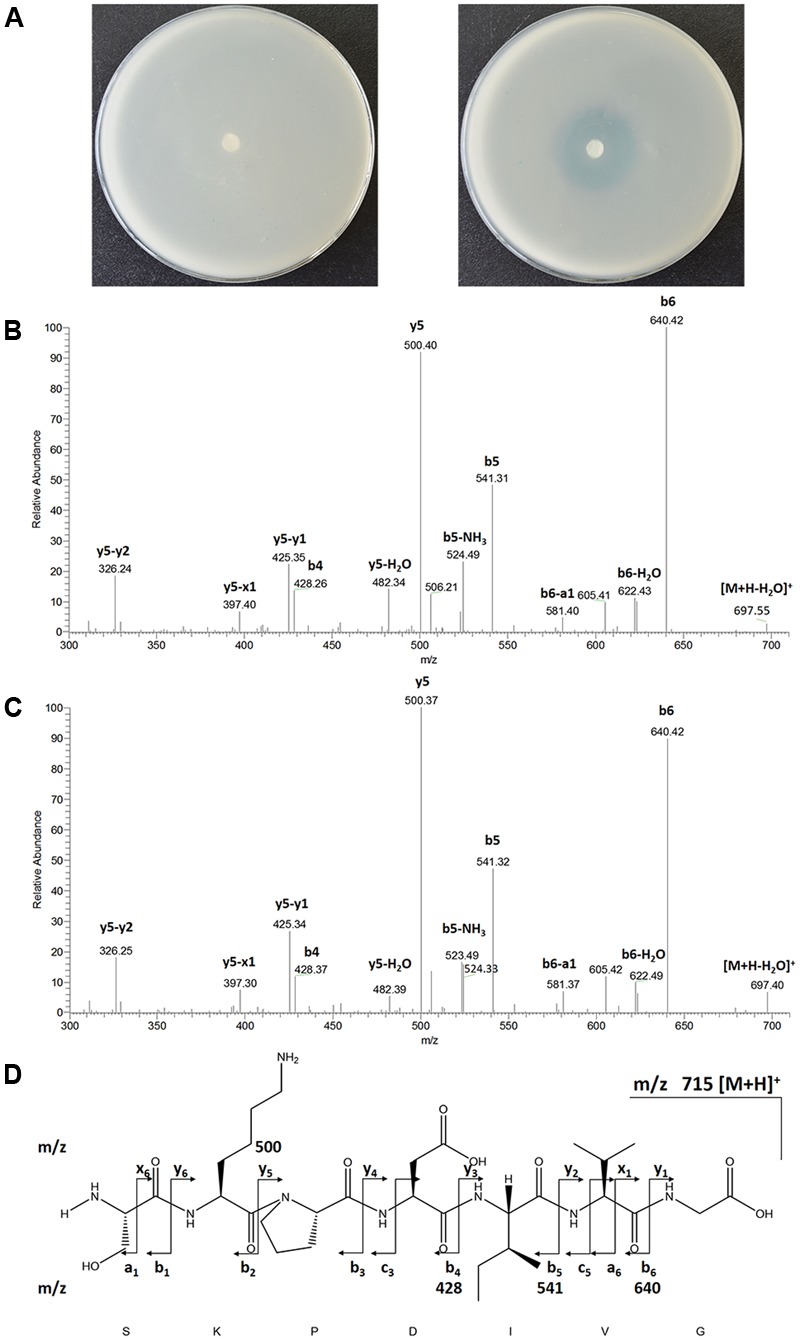
Agar plate assay and MS/MS analysis of the NprX peptide. **(A)** Paper disk diffusion assay of NprX peptide extracted by a Waters Sep Pak C18 cartridge by using the reporter strain *Bacillus thuringiensis* 407 Cry^-^ (*nprA′Z* ΔRX [pHT304-R]). The left plate is an extract of BEP medium (control), and the right plate is an extract of the culture supernatant from strain S51107. **(B)** MS/MS spectra fragmentation of the SKPDIVG synthetic heptapeptide (standard substance). **(C)** MS/MS spectra fragmentation of NprX peptide extracts from strain S51107 culture. **(D)** Theoretical fragmentation of the SKPDIVG peptide.

To identify exactly the structure of the NprX peptide, the solid phase extract of the cell-free supernatant of strain S51107 was analyzed with high liquid chromatography coupled linear ion trap quadrupole mass spectrometer (LTQ). The solid phase extracts were pre-assayed by using the reporter strain. As shown in **Figure 3A**, the extracts of strain S51107 (right agar plate) enabled the reporter strain to appear blue and the corresponding control medium (left agar plate) did not, indicating that the extracts of strain S51107 contained NprX. The extracted ion chromatogram of *m/z* at 715.5 (the *m/z* of ion [M+H]^+^ of NprX peptide) shared the same retention time at 2.8 min, with a sharp peak for the standard SKPDIVG peptide (Supplementary Figure S5). The MS/MS mass spectra fragmentations of standard SKPDIVG (**Figure 3B**) and NprX from strain S51107 (**Figure 3C**) were in accord with the theoretical mass spectrum analysis (**Figure 3D**). These analyses confirmed that strain S51107 produces NprX heptapeptide SKPDIVG as a signaling peptide.

### Disruption of *nprR-nprX* Genes in *Bacillus* sp. Strain S51107 Affects the Strain’s Algicidal Activity against *M. aeruginosa*

To investigate the role of the NprR-NprX quorum-sensing system in the algicidal activity of *Bacillus* sp. strain S51107, a mutant strain disrupted *nprR-nprX* gene was constructed via homologous recombination. The PCR analysis and sequencing results verified that the *nprR-nprX* gene was successfully disrupted in the *nprR-nprX* mutant (Supplementary Figure S6). Moreover, expression of the NprX signaling peptide was validated by using the reporter strain for the wild type and all mutant strains. The results showed that only the wild type strain and the complemented strain *nprR-nprX* [pHT304-RX] produced NprX, turning the reporter strain blue (Supplementary Figure S7). However, the negative agar plate results showed that the *nprR-nprX* mutant, *nprR-nprX* [pHT304-R] mutant and *nprR-nprX* [pHT304] did not produce NprX.

**FIGURE 4:**
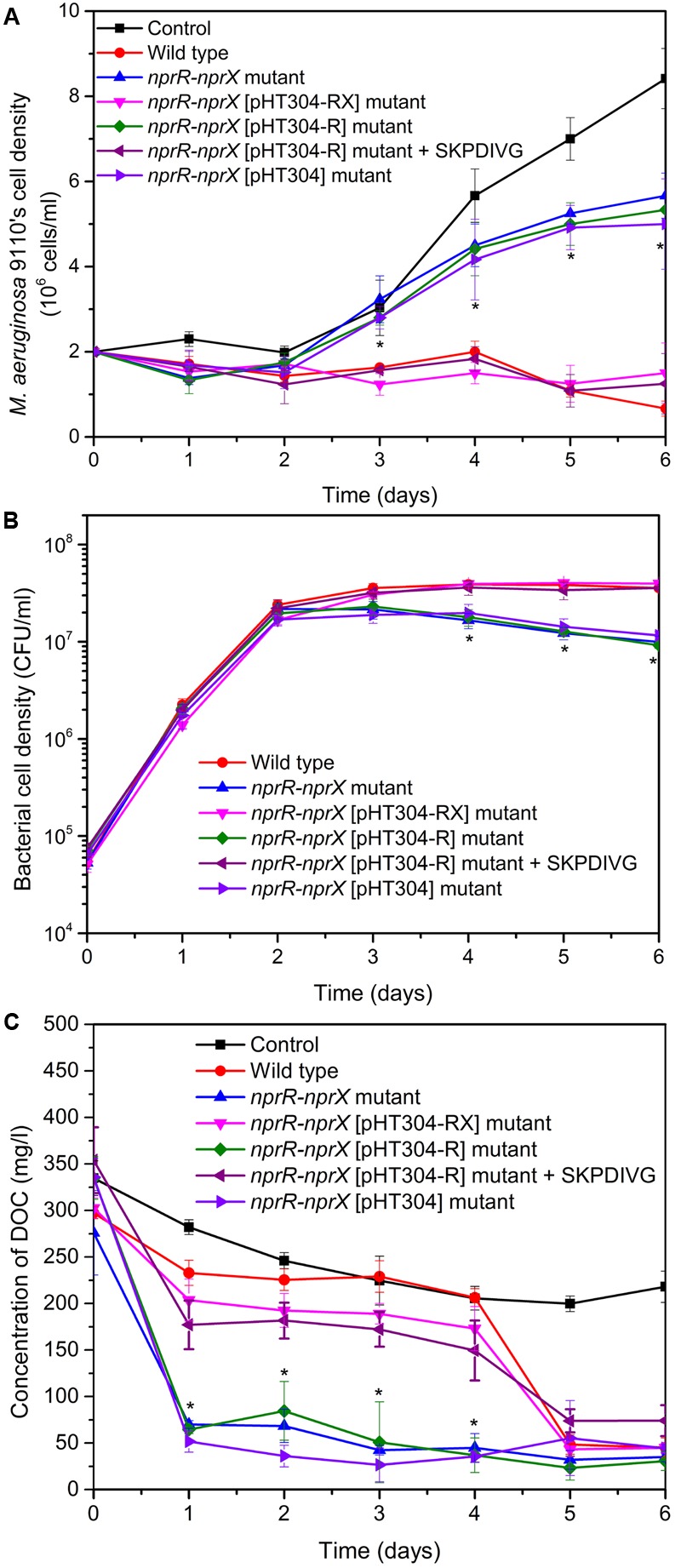
Dynamics of the cell densities of *M. aeruginosa* 9110 **(A)** and the algicidal strains **(B)** and the concentration of dissolved organic carbon (DOC) **(C)** during the algicidal process of *Bacillus* sp. strain S51107 and its mutants against *M. aeruginosa* 9110. The data are shown as the averages of three independent experiments (error bars are the SD from the mean values). The values of group B marked with ^∗^ were significantly different (*P* < 0.01) from those of group A at the same time point. Group A: wild type, *nprR-nprX* [pHT304-RX] mutant, *nprR-nprX* [pHT304-R] mutant + SKPDIVG; group B: *nprR-nprX* mutant, *nprR-nprX* [pHT304-R] mutant, *nprR-nprX* [pHT304] mutant. For the control, BEP medium was added to *M. aeruginosa* 9110 cultures instead of the bacterial culture.

As shown in **Figure 4**, the co-cultures of *M. aeruginosa* 9110 with wild type strain S51107 or its mutants displayed two variation tendencies in the above parameters; thus, we classified them into two groups (group A: co-cultures in the presence of wild type strain S51107, *nprR-nprX* [pHT304-RX] mutant, and *nprR-nprX* [pHT304-R] mutant supplemented with 5 μM SKPDIVG peptide; group B: co-cultures in the presence of *nprR-nprX* mutant, *nprR-nprX* [pHT304-R] mutant, and *nprR-nprX* [pHT304] mutant).

As shown in **Figure 4A**, the cell densities of *M. aeruginosa* 9110 in the co-cultures of group B were significantly higher than those of group A after day 3, indicating that the algicidal activities of the mutants with a disrupted NprR-NprX QS system were significantly (*P* < 0.01) weakened relative to those of wild type strain S51107 and complemented mutants after 3 days of co-cultivation. The bacterial cell density of all strains increased during the first 2 days to approximately 2 × 10^7^ CFU/ml (**Figure 4B**), meanwhile, the DOC declined sharply (**Figure 4C**). After 3 days of co-cultivation, the cell densities of bacterial strains in the co-cultures of group A were significantly (*P* < 0.01) higher than those of group B, except for day 3 (*P* < 0.05). In particular, from days 3 to 6, the population of the strains in the co-cultures of group A remained stable, whereas the population of the strains in the co-cultures of group B decreased gradually. From days 2 to 4, the DOC in the co-cultures of group A was higher than that of group B (*P* < 0.01). On days 5 and 6, the DOC in the co-cultures of group A decreased to the level of group B approximately 45 mg/l (**Figure 4C**). These findings indicate that the NprR-NprX QS system is responsible for modulating the algicidal activity of *Bacillus* sp. strain S51107 against *M. aeruginosa* 9110.

### Purification of Low-Molecular-Weight Algicidal Compounds and the Effects of the NprR-NprX QS System on the Concentration of Low-Molecular-Weight Algicidal Compounds

#### Extraction, Purification, and Identification of Low-Molecular-Weight Algicidal Compounds

**FIGURE 5:**
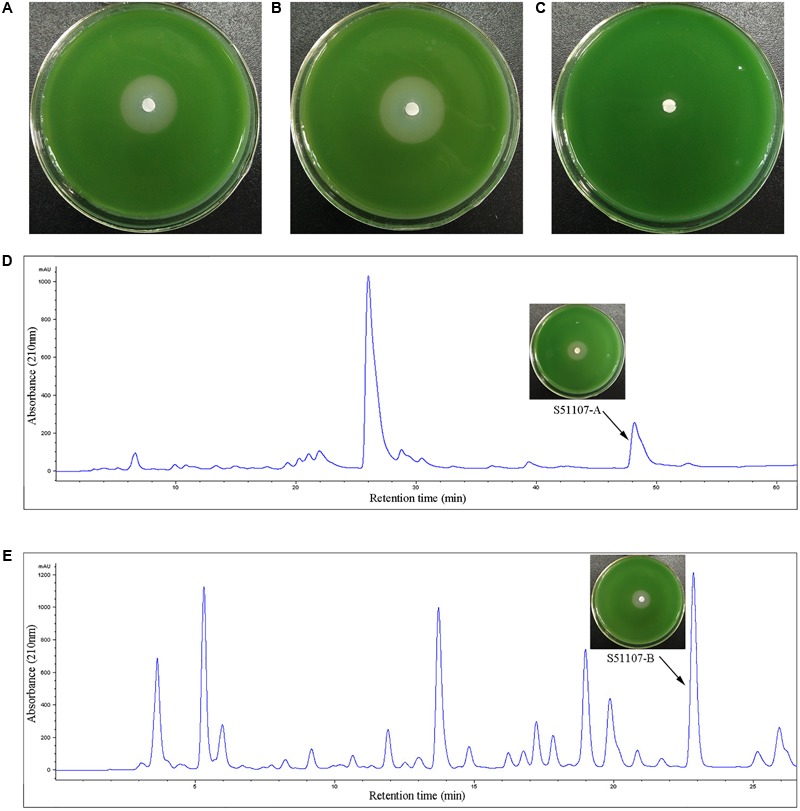
Algicidal effects of silica gel chromatography fractions on cyanobacterial-lawn and high performance liquid chromatography (HPLC) separation of the algicidal fractions S2 and S5. **(A–C)** indicate the cyanobacterial-lawn results of algicidal fraction S2, S5 and control (other fractions), respectively. HPLC was performed on a Zorbax^®^ Bonus-RP column (4.6 mm × 250 mm, 5 μm). The fraction S2 was eluted with a linear gradient of MeOH/H_2_O from 5 to 47% (vol/vol) for 60 min at a flow rate of 1.0 ml/min, yielding pure S51107-A (retention time = 47.5–49.0 min) **(D)**. The fraction S5 was purified with MeOH/H_2_O from 10 to 60% for 40 min yielded pure S51107-B (retention time = 22.6–23.2 min) **(E)**. The arrows denote the algicidal effect of fraction on the cyanobacterial-lawn.

The ethyl acetate extraction from the cell free supernatant of strain S51107 exhibited prominent algicidal activity. We found two fractions (S2 and S5) with algicidal activity after silica gel chromatography separation (**Figures 5A–C**). High-performance liquid chromatography (HPLC) purification resulted in two constituents, S51107-A and S51107-B (**Figures 5D,E**), with algicidal effects against *M. aeruginosa*.

**FIGURE 6:**
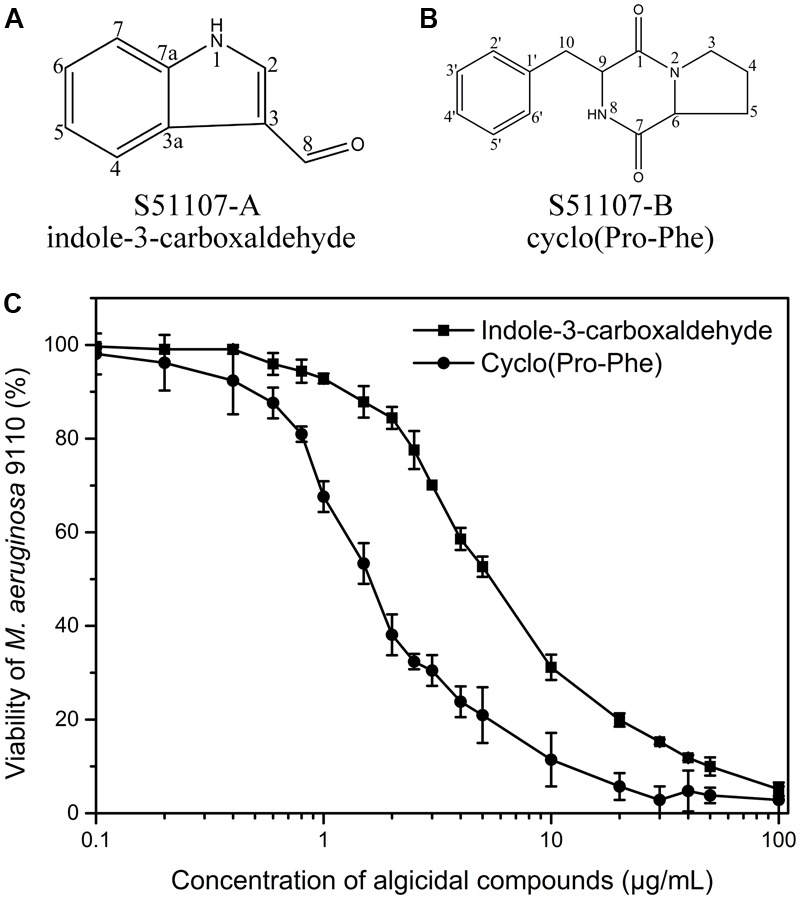
Molecular structures of identified algicidal compounds **(A,B)** and algicidal effects of the compounds against *M. aeruginosa* 9110 after 24 h exposure **(C)**.

The high-resolution electrospray ionization mass spectra (ESI-MS) of S51107-A and S51107-B yielded molecular ions at 144.0450 *m/z* (M-H)^-^ and 245.1296 *m/z* (M+H)^+^ (Supplementary Figure S8), respectively, and probable molecular formulas of C_9_H_7_NO and C_14_H_16_N_2_O_2_, respectively. The mass spectra of the electron ionization mass spectra (EI-MS) were compared with those in the NIST/EPA/NIH Mass Spectra library, thus indicating that the algicidal compounds S51107-A and S51107-B were indole-3-carboxaldehyde and 3-benzyl-hexahydro-pyrrolo[1,2-*a*]pyrazine-1,4-dione [“cyclo(Pro-Phe)” for short], respectively, with a high similarity index (>950) (Supplementary Figure S9). The EI-MS mass spectra of S51107-A (Supplementary Figure S9A) and S51107-B (Supplementary Figure S9D) were consistent with those of standard indole-3-carboxaldehyde (Supplementary Figure S9B) and cyclo(Pro-Phe) (Supplementary Figure S9E), respectively. Furthermore, the structures of S51107-A and S51107-B (**Figures 6A,B**, respectively) were confirmed by ^1^H-NMR and ^13^C-NMR data (Supplementary Table S4).

The EC_50_ values (half maximal effective concentration) of standard indole-3-carboxaldehyde and cyclo(Pro-Phe) against *M. aeruginosa* 9110 were estimated as 6.55 and 1.85 μg/ml, respectively, from dose response curves (**Figure 6C**), which indicated that cyclo(Pro-Phe) exhibited a higher algicidal activity than indole-3-carboxaldehyde.

#### Dynamics of the Concentration of Low-Molecular-Weight Algicidal Compounds during the Algicidal Process in Co-culture

To investigate whether the low-molecular-weight algicidal components, indole-3-carboxaldehyde and cyclo(Pro-Phe), are regulated by the NprR-NprX QS system, the concentrations of algicidal compounds were tested during the algicidal process against *M. aeruginosa*. There were no significant differences in the concentrations of indole-3-carboxaldehyde and cyclo(Pro-Phe) between the group A and group B co-cultures (Supplementary Figure S10). Moreover, no obvious differences were found in the productions of algicidal compounds between the wild type strain and the mutant strains, which were pure-cultured in BEP liquid medium, respectively (data not shown). These results suggested that two low-molecular-weight algicidal compounds were not modulated by the NprR-NprX QS system and that strain S51107 might excrete additional types of algicidal substances.

### The NprR-NprX QS System Regulates the Production of High-Molecular-Weight Algicidal Compounds of *Bacillus* sp. Strain S51107 against *M. aeruginosa*

**FIGURE 7:**
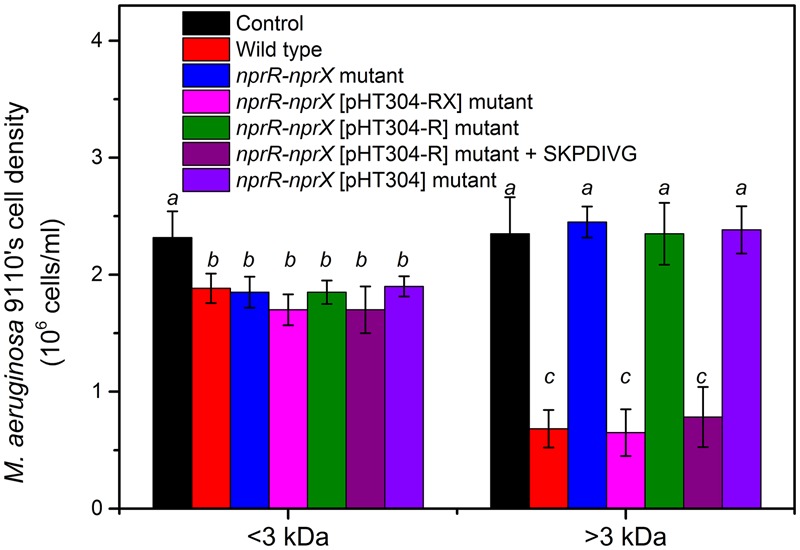
Algicidal effects of size-fractionated (>3 and <3 kDa) cell-free supernatant of BEP medium (control), wild type strain S51107, *nprR-nprX* mutant, *nprR-nprX* [pHT304-RX] mutant, *nprR-nprX* [pHT304-R] mutant, *nprR-nprX* [pHT304-R] mutant + SKPDIVG and *nprR-nprX* [pHT304] mutant on *M. aeruginosa* 9110. The cell densities of *M. aeruginosa* 9110 was counted after 48 h. As a control, BEP medium was fractionated and the algicidal activities of the filtrates were monitored. The data are the averages of three independent experiments (error bars are SD from mean values). Different letters indicate statistically significant differences (*P* < 0.05).

To investigate the NprR-NprX QS-mediated algicidal compounds of strain S51107, size fractionation experiments were conducted using centrifugal filter units with a molecular weight cut off of 3 kDa. As shown in **Figure 7**, the >3 kDa fraction of the cell-free filtrate from mutants with an inactivated-QS system had significantly lower algicidal activity than that of wild type strain S51107 and complemented mutants (*P* < 0.01). However, there were no significant differences in the algicidal activities of the <3 kDa fraction of the cell-free filtrate between wild type strain S51107 and its derived mutants. It indicated that the NprR-NprX QS system in *Bacillus* sp. strain S51107 regulated the production of extracellular high-molecular-weight algicidal compounds (>3 kDa) and the production of <3 kDa algicidal compounds was not influenced by QS system.

The >3 kDa fraction of wild type strain S51107 exhibited the algicidal activity of 71% (**Figure 7**). The concentration of 0.11 μg/mL of indole-3-carboxaldehyde and 0.75 μg/mL of cyclo(Pro-Phe) were detected in the <3 kDa fraction. When this concentration of two compounds was added into *M. aeruginosa* together, it showed comparable algicidal activity to that of the <3 kDa fraction (data not shown). It indicated that indole-3-carboxaldehyde and cyclo(Pro-Phe) might contribute to the algicidal activity of the <3 kDa fraction (about 20% of activity, **Figure 7**).

## Discussion

NprR (Neutral Protease Regulatory), a member of the RRNPP family, has been found in *B. cereus* group, which includes *B. thuringiensis, B. cereus, B. mycoides, B. pseudomycoides, B. weihenstephanensis*, and *B. anthracis* (Perchat et al., 2011). The activity of NprR depends on its cognate signaling peptide, NprX, which is exported outside the cell for processing into an active heptapeptide and is subsequently reimported into the cell. NprX specifically interacts with NprR, thus allowing the NprR-NprX complex to bind to its DNA target sites and activate gene expression. In *B. thuringiensis*, transcriptomic analysis has revealed that the NprR-NprX QS system controls the expression of at least 41 genes that encode neutral protease (NprA), degradative enzymes and other proteins; is involved in nutrient supply, stress and antibiotic resistance; and controls the synthesis of kurstakin, a non-ribosomal peptide (Dubois et al., 2013). The NprR-NprX QS system was found in algicidal bacteria *Bacillus* sp. strain S51107 in the current research. Earlier studies have demonstrated that homoserine lactone and alkylquinolone mediated quorum sensing are associated with regulation of the algicidal activity of Gram-negative bacteria (Guo et al., 2016; Harvey et al., 2016). For Gram-positive algicidal bacteria, research has shown that their algicidal effects depend on bacterial cell density (Wang et al., 2013; Jiang et al., 2014; Shao et al., 2014). Whether the quorum signaling mechanism is involved in the modulation of the algicidal activity of Gram-positive bacteria remains unclear. The present findings demonstrate that the NprR-NprX QS system regulates the algicidal function of the Gram-positive algicidal bacteria *Bacillus* sp. strain S51107 against *M. aeruginosa*.

In this study, *Bacillus* sp. strain S51107 was found to produce two low-molecular-weight algicidal compounds, indole-3-carboxaldehyde and cyclo(Pro-Phe). This study provides the first reported evidence that indole-3-carboxaldehyde, an indole derivative from amino acid metabolism, is an algicidal compound with activity against *M. aeruginosa*. Other indole derivatives with algicidal activity have been found. In addition to 3-methylindole (Guo et al., 2016) and isatin (Li et al., 2014), gramine is a suppressor of energy transfer in photosystem II (PSII) of cyanobacterial species (Laue et al., 2014), and tryptamine and tryptoline kill *Microcystis* sp. by promoting ROS production and inhibiting antioxidant synthesis (Zhang et al., 2016). Cyclo-(Pro-Phe), a cyclic dipeptide, was first identified to have algicidal activity against *M. aeruginosa* in this study. Other cyclic dipeptides with algicidal activity have been reported, such as cyclo(Pro-Gly) (Li et al., 2014; Li Z. et al., 2015; Lin et al., 2016; Tan et al., 2016), cyclo(Pro-Val) (Li Z. et al., 2015), cyclo(Pro-Leu) and cyclo(4-OH-Pro-Leu) (Guo et al., 2015), and cyclo(Gly-Phe) (Guo et al., 2016). Knockout of the genes of NprX signaling peptide and its cognate NprR regulator in strain S51107 reveal that synthesis of indole-3-carboxaldehyde and cyclo-(Pro-Phe) are not regulated by the NprR-NprX QS system. This finding is distinct from a previous report indicating that the C4-HSL-mediated QS system of Gram-negative *Aeromonas* sp. strain GLY-2107 positively regulates the synthesis of the algicidal compound 3-methylindole and negatively regulates that of cyclo(Gly-Phe) (Guo et al., 2016). Although there was no clear difference in the concentrations of two low-molecular-weight algicidal compounds between the wild type strain S51107 and derived mutants during the algicidal process, the *nprR-nprX* mutant showed a dramatic decrease in algicidal activity. These results indicate that the two low-molecular-weight algicidal compounds might play minor roles in the algicidal process.

*Bacillus* sp. strain S51107 also produced high-molecular-weight (>3 kDa) algicidal substances. The deletion of the *nprR-nprX* genes led to a significant decrease in the algicidal activity of cell-free filtrate with a molecular weight fraction >3 kDa, as compared with the results for the wild type strain. Together with the significant decrease in the algicidal activity of cell-free supernatant from strain S51107 after heat treatment, these results suggest that the NprR-NprX QS system might modulate the secretion of algicidal proteins. Proteins in general have been reported as candidate algicidal substances. For example, Afi et al. (1996) have found that the degradation of *Chlorella vulgaris* by the bacteria *P. oleovorans* and *Flavobacterium aquatile* is probably mediated by extracellular enzymes. Lee et al. (2000) have found that *Pseudoalteromonas* sp. strain A28 produces an active serine protease algicidal to the diatom *Skeletonema costatum*. Chen et al. (2011) have found that L-amino acid oxidase from *Aquimarina* sp. has algicidal activity against *M. aeruginosa*.

Notably, AHL-mediated QS of the Gram-negative γ-proteobacterium strain MS-02-063 is involved in the production of a red algicidal pigment (Nakashima et al., 2006). The C4-HSL-mediated QS system of Gram-negative *Aeromonas* sp. modulates the production of two algicidal compounds that are primarily responsible for the algicidal function (Guo et al., 2016). However, a Gram-negative algicidal bacterium, *Kordia algicida*, releases a quorum-sensing-dependent protease against *S. costatum* without any AHL (Paul and Pohnert, 2011). Gram-negative *P. piscicida* releases HHQ, a precursor of another class of QS signaling molecules (alkylquinolone), thereby inducing the death of *E. huxleyi* through an unknown algicidal mechanism (Harvey et al., 2016). In the present study, the NprR-NprX QS system of Gram-positive *Bacillus* sp. strain S51107 modulated the high-molecular-weight algicidal substances (>3 kDa). Therefore, we can find that different types of QS systems are involved in the algicidal activity and the QS-regulated algicidal mechanisms of algicidal bacteria may be more diverse and complicated.

The DOC derived from phytoplankton exudation or cell lysis is a major energy source that stimulates heterotrophic prokaryote growth (Sarmento and Gasol, 2012). The growth rate of strain S51107 did not significantly differ from that of its derived mutants in BEP-rich medium (Supplementary Figure S11). However, during the algicidal process, the cell densities of wild type strain S51107 and the genetic or chemical complementary *nprX* and *nprR-nprX* mutants (group A) were significantly higher than those of the *nprX* and *nprR-nprX* mutants (group B) after day 3 of co-cultivation (**Figure 4B**). This result might have been because after 2 days of co-cultivation, the DOC arising from the lysis of cyanobacterial cells in group A caused more bacterial cells to multiply, and the higher concentration of bacteria in group A further resulted in more cyanobacteria to be lysed and more DOC released. After 4 days of co-cultivation, the decreased DOC concentration in group A co-culture was probably the result of a more rapid depletion rate of DOC by the algicidal bacteria than the production rate of DOC from cyanobacterial exudation or cell lysis.

The present study demonstrates that NprR-NprX QS of Gram-positive *Bacillus* sp. S51107 modulates the algicidal activity of the strain, and the extracellular high-molecular-weight algicidal substances (>3 kDa) are primarily responsible for the algicidal activity. The two non-QS-regulated low-molecular-weight algicidal compounds, indole-3-carboxaldehyde and cyclo-(Pro-Phe), relatively play minor roles. *Bacillus* sp. S51107 induced the mortality of *M. aeruginosa* 9110 by production of indole-3-carboxaldehyde, cyclo-(Pro-Phe) and the QS-regulated algicidal compounds (>3 kDa) simultaneously. Moreover, this is the first evidence of algicidal substance production being regulated by QS in Gram-positive bacteria. Although the extracellular high-molecular-weight algicidal substances is unclear, our data improve understanding of the interactions between cyanobacteria and indigenous algicidal bacteria through chemical signal molecules and may aid in the design and optimization of strategies to control harmful algae blooms.

## Author Contributions

LW and HY designed experiments. LW, XG, and XL performed the experiments. LW and HY analyzed the data. LW and HY wrote the manuscript.

## Conflict of Interest Statement

The authors declare that the research was conducted in the absence of any commercial or financial relationships that could be construed as a potential conflict of interest.
