# Supplementary material for: NprR-NprX Quorum-Sensing System Regulates the Algicidal Activity of Bacillus sp. Strain S51107 against Bloom-Forming Cyanobacterium Microcystis aeruginosa
Source: Front Microbiol. 2017 Oct 11;8:1968. doi: 10.3389/fmicb.2017.01968 (PMC5641580; doi:10.3389/fmicb.2017.01968)
Supplement: Supplementary file 1 [file Presentation_1.PDF]

Supplementary Material

NprR-NprX quorum-sensing system regulates the algicidal activity of *Bacillus* sp. strain S51107 against bloom-forming cyanobacterium *Microcystis aeruginosa*

Lishuang Wu[1], Xingliang Guo[1], Xianglong Liu[1], Hong Yang[1]*

[1]State Key Laboratory of Microbial metabolism, School of Life Science & Biotechnology, Shanghai Jiao Tong University, Shanghai, 200240, P.R. China

***Correspondence:** Hong Yang: hongyang@sjtu.edu.cn

SUPPLEMENTARY MATERIALS AND METHODS

Determination of bacterial cell density

The cell densities of bacterial strains were counted by colony-forming units (CFU) method on BEP agar plates. The sample to be detected was serially diluted to appropriate concentration with sterile BG11 medium according to 10-fold dilution method. An aliquot (100 μl) of dilutions was spread onto the BEP plates (1.5% agar, w/v). The plates were cultured for 24 h at 30 ℃, and the number of colony was immediately counted. The bacterial population was calculated by the following equation: bacterial population = $N_c \times M_d \times 10$, where N_c and M_d represent the number of colony and dilution multiple, respectively.

Identification of NprX peptide by LC-MS/MS

The extracts were purified on a C18 RP-column (Cortecs®C18, 2.7 μm, 2.1 × 100 mm, Waters) with a flow rate of 0.25 ml/min. The column was eluted by using the mobile phase containing water (solution A) and acetonitrile (solution B). The gradient elution procedure was as follows: 0-3 min 5% B, 3-11 min 5% B-100% B, 11-11.5 min 100% B-5% B, 11.5-15 min 5% B. The MS scanned in ESI source in positive mode, and the product was scanned at *m/z* 715.5 for NprX peptide with the following parameters: source voltage, 4 kV; capillary voltage, 24.0 V; capillary temperature, 320 ℃; sheath gas flow rate, 10 arb; sweep gas flow rate, 0 arb. Mass spectra were acquired from 195 and 1000 *m/z* with the collision energy at 35 eV for fragment ion scanning. Data acquisition, data processing, and instrument control were performed using an Xcalibur 2.3.1 software (Thermo Fisher, USA).

Isolation and purification of bacterial algicidal compounds

The isolation and purification of algicidal compounds was accomplished by using our previous method (Lin et al., 2014) with some modifications. Strain S51107 was grown in an Erlenmeyer flask with BEP liquid medium and cultivated at a shaking speed of 200 rpm at 28°C for 24 h. The cell-free supernatant was extracted with an equal volume of ethyl acetate (EtOAc) three times. The combined EtOAc extracts were evaporated to dryness and chromatographed over a silica gel column with different solvents of increasing polarity (petroleum ether/acetone followed by $CHCl_3$/MeOH) thus yielding twelve fractions on the basis of TLC pattern. TLC was performed on normal-phase silica gel sheets and visualized by UV light at 254 nm before spraying with vanillin-sulfuric reagent and heating. The algicidal sub-fractions from the silica-gel column were purified with a reversed-phase C18 column (Zorbax® Bonus-RP column, 4.6 mm × 250 mm, 5 μm, Agilent, USA) on the HPLC system (1260 Infinity, Agilent, USA) with a UV-Vis detector (G1314F, Agilent, USA) at 210 nm. The algicidal activity of each fraction was examined by using the cyanobacterial-lawn method (Tian et al., 2012). Finally, the purified algicidal compounds were collected for structural elucidation.

Identification and quantification of algicidal compounds

The identification of algicidal compounds by combining the high-resolution electrospray ionization mass spectrum (HR-ESI-MS), electron ionization mass

spectrum (EI-MS), and nuclear magnetic resonance (NMR) and the quantification of algicidal compounds during the lysing process were conducted as described in our previous work (Li et al., 2014; Guo et al., 2016) with slight modifications.

HR-ESI-MS was conducted on ultra-performance liquid chromatography coupled with time-of-flight mass spectrometer (UPLC-TOF-MS, Agilent Technologies HPLC 1290-MS 6230, USA). The HPLC condition was as following: Agilent Eclipse XDB-C18 column (5 μm, 4.6 × 150 mm), methanol-water linearity gradient elution (5%-100%, vol/vol) at flow rate of 0.4 ml/min and wavelength of 210 nm. The TOF mass spectrometer with ESI source was performed in positive or negative ionization mode (ESI^+ or ESI^-) with the following parameters: capillary voltage, 4000 V (ESI^+) or 3500 V (ESI^-); nozzle voltage, 200 V; skimmer voltage, 65 V; fragmentor voltage, 105 V; sheath gas temperature and flow, 350°C and 11 l/min; drying gas temperature and flow, 325°C and 8 l/min; nebulizer pressure, 35 psig. The data were collected between 100 and 1,700 *m*/*z*. The mass data were analyzed by the Agilent MassHunter Workstation Data Acquisition Software version 6.0.

EI-MS was carried on gas chromatography coupled with an electron ionization mass spectrometer (Agilent 6850/5975C) under HP5-MS column (30-m capillary column, 0.25 μm film thickness) and using highly pure helium as the carrier gas (flow rate =1 ml/min). The GC conditions were as follows: initial oven temperature 50°C for 2 min, increased to 100°C at the rate of 10°C/min, then held at 100°C for 2 min, and followed by an increase of 10°C/min to 300°C and held for 5min. The injector and interface temperature was set at 280°C. Mass spectra were operated in electron ionization mode with energy of 70 eV and analyzed in full scan mode over a range of *m*/*z* 29-1000. The MS data were identified with the NIST/EPA/NIH Mass Spectra library (Version 2.0). NMR spectroscopy were recorded on a 600 MHz Bruker Avance III spectrometer (Switzerland) at 295 K (^{1}H-NMR at 600 MHz, ^{13}C-NMR at 151 MHz). The algicidal compounds S51107-A and S51107-B were dissolved in CD_3OD.

The method for the quantification of algicidal compounds was performed as our previous work (Guo et al., 2016) using ultra performance liquid chromatography coupled with mass spectrometry (UPLC-MS) (Agilent Technologies HPLC 1290-MS 6230, USA) in positive mode. The HPLC condition was as following: Agilent Eclipse XDB-C18 column (5 μm, 4.6 × 150 mm) and methanol-water linearity gradient elution. Data was acquired and processed by the LC/MS Qualitative Analysis B.06.00 software (Agilent Technologies, USA).

Dose response bioassays of indole-3-carboxaldehyde and cyclo-(Pro-Phe) against *M. aeruginosa* 9110

The algicidal activities of indole-3-carboxaldehyde and cyclo-(Pro-Phe) against *M. aeruginosa* 9110 were investigated by using a dose-response protocol according to the method described in our previous study (Guo et al., 2016) with some modifications. Ten microliters of serial concentration stock solutions (0.05, 0.1, 0.2, 0.3, 0.4, 0.5, 0.75, 1, 1.25, 1.5, 2, 2.5, 5, 10, 15, 20, 25, and 50 mg/ml) of each compound dissolved in dimethyl sulfoxide (DMSO) was added to 4.99 ml of log-phase *M. aeruginosa* 9110 to form final concentrations of 0.1, 0.2, 0.4, 0.6, 0.8, 1, 1.5, 2,2.5, 3, 4, 5, 10, 20, 30, 40,

50, and 100 μg/ml, respectively. An equal volume of DMSO was used as a control. Yi et al. have demonstrated that 0.3% (vol/vol) of DMSO does not affect the growth of *M. aeruginosa* (Yi et al., 2012). The viability of *M. aeruginosa* 9110 was tested after 24 h of exposure and calculated by the following equation: $V\,(\%) = D_{t\text{-}treatment}/D_{t\text{-}control} \times 100$, where $D_{t\text{-}treatment}$ and $D_{t\text{-}control}$ are the cell densities of cyanobacteria with the treatment and control, respectively, and t is the incubation time. The EC_{50} values were calculated through probit analysis from the dose-response curves (Zhang et al., 2013).

SUPPLEMENTARY FIGURES

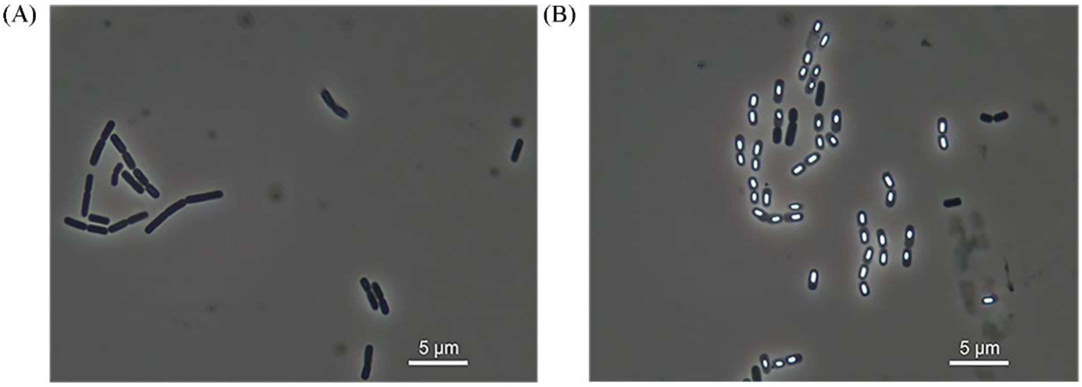

Supplementary Figure S1. Phase-contrast micrographs of *Bacillus* sp. strain S51107. Phase-contrast micrographs of the cell morphology of *Bacillus* sp. S51107 (×1000 magnification) after 24h (A) and 48h (B) of cultivation on BEP agar.

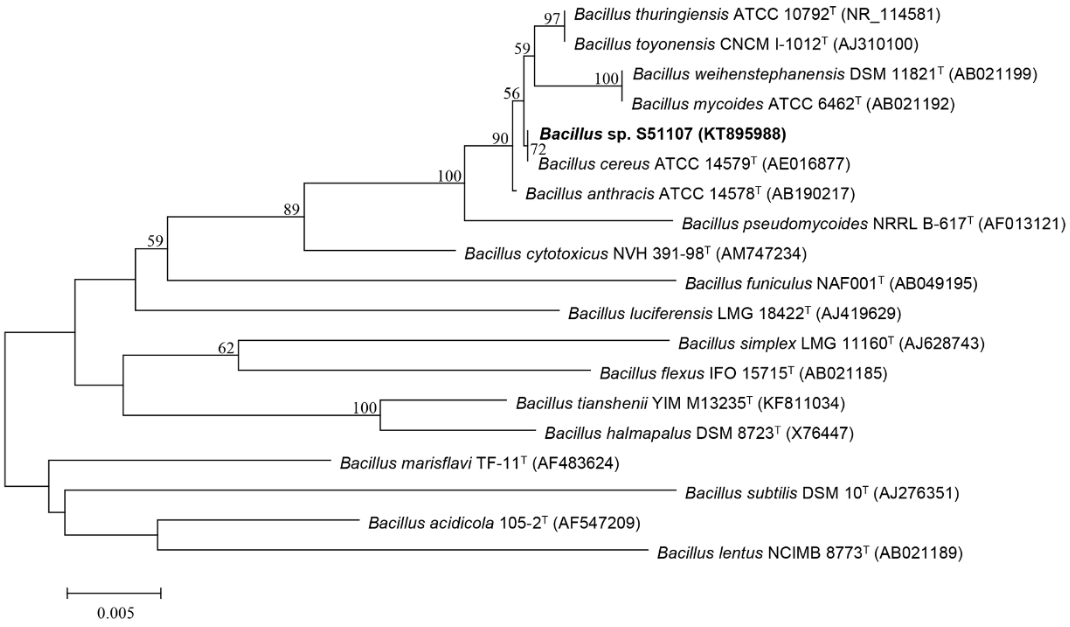

Supplementary Figure S2. Neighbour-joining phylogenetic tree of strain S51107 (in bold) and closely related species of the *Bacillus* genus based on 16S rRNA gene sequences. One thousand bootstrap replicates were performed for statistical validation (numbers in the nodes indicate the percentage bootstrap values which were above 50%). GenBank accession numbers are showed in parentheses after strains name. The scale bar denotes 0.005 substitution per nucleotide position.

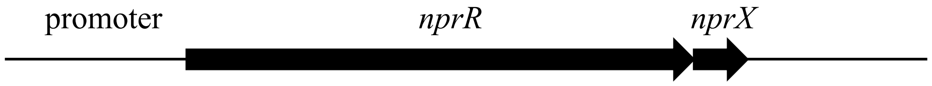

Supplementary Figure S3. Schematic view of the *nprR* and *nprX* genes in *Bacillus* sp. strain S51107.

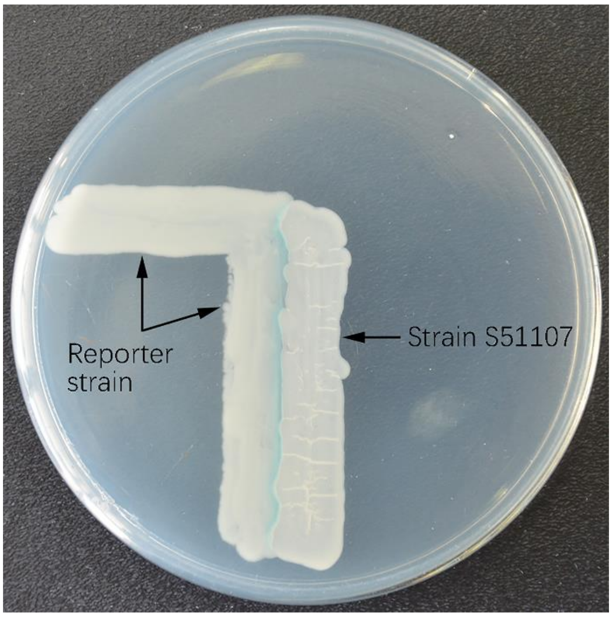

Supplementary Figure S4. Detection of the NprX signaling peptide from strain S51107 by the reporter strain *B. thuringiensis* 407 Cry$^-$ (*nprA'Z* ΔRX [pHT304-R]).

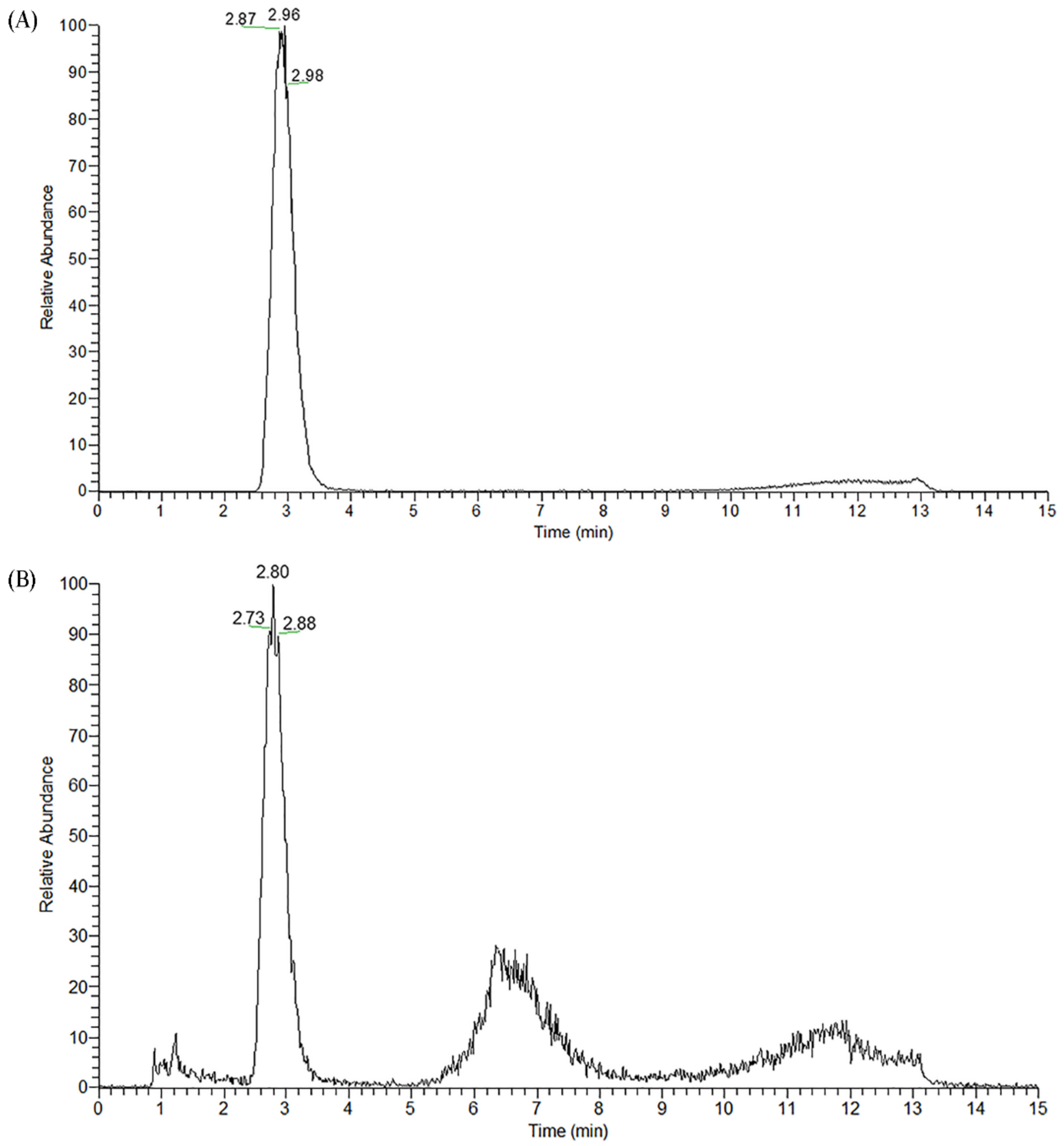

Supplementary Figure S5. MS/MS analysis of standard of SKPDIVG synthetic heptapeptide and NprX extracted from *Bacillus* sp. strain S51107 culture. Extracted ion chromatography with *m/z* 715.50 from total ion current chromatography of MS/MS spectra from standards (A) and extractive of *Bacillus* sp. strain S51107 culture (B).

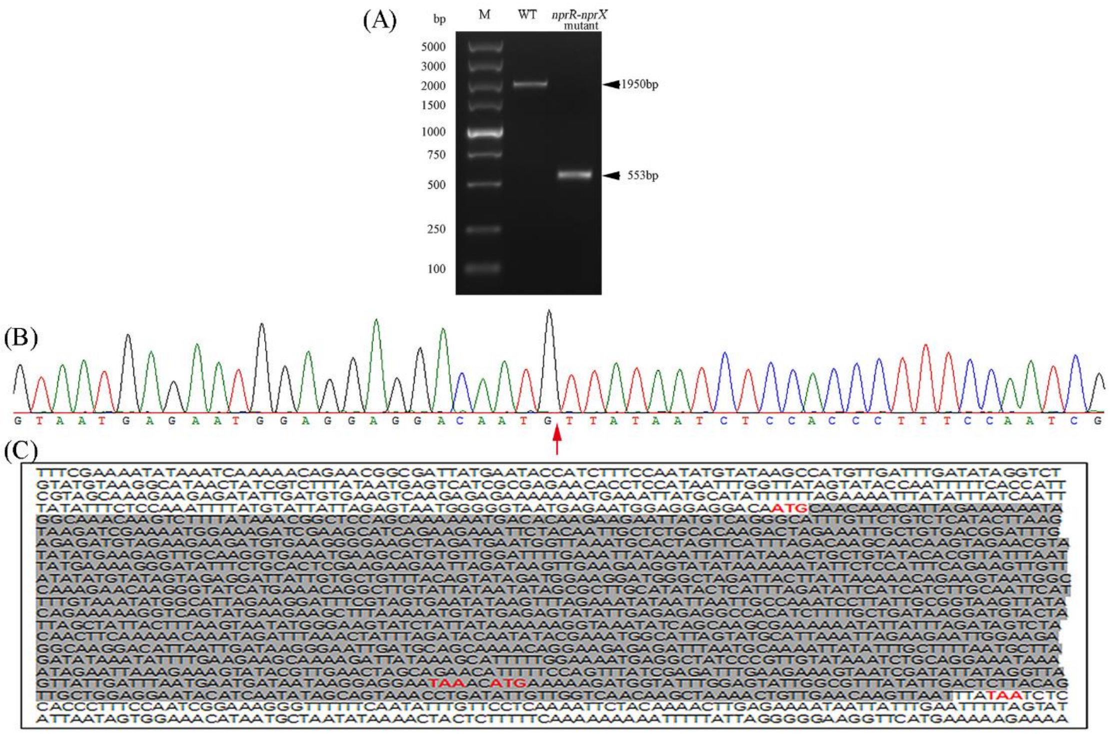

Supplementary Figure S6. Confirmation of *nprR-nprX* mutant by PCR analysis and sequencing. (A) PCR amplification was performed with primers Npr1E-F and NprR-2, and the products were analyzed by1.5% agarose gel electrophoresis. Lane M, DNA molecular weight marker; lane WT, PCR product from chromosomal DNA of wild type strain S51107; lane *nprR-nprX* mutant, PCR product from chromosomal DNA of *nprR-nprX* mutant strain. (B) Sequencing results of the DNA sequence near the deletion mutation site (indicated in red arrow) in *nprR-nprX* mutant strain. (C) The sequence near the *nprR-nprX* gene in wild type strain S51107. The region shaded gray was deleted in the *nprR-nprX* mutant strain. The start codon and stop codon of *nprR* (upstream) and *nprX* (downstream) genes were in red color.

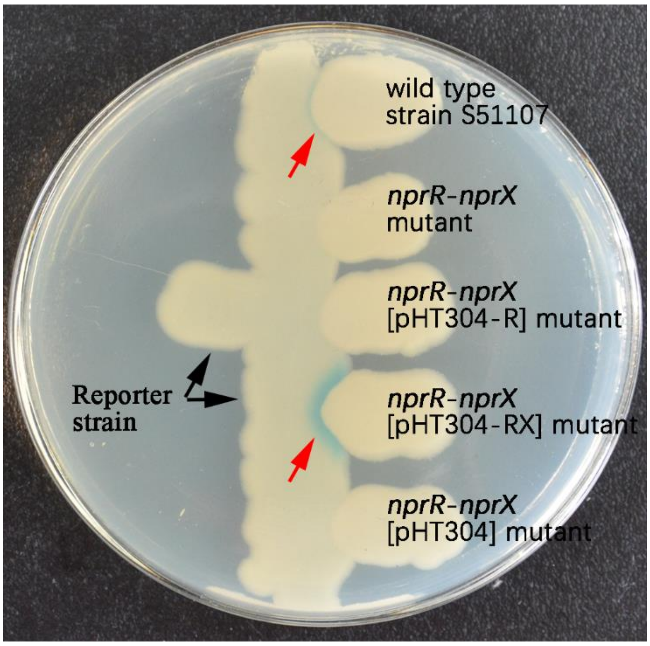

Supplementary Figure S7. Detection of signaling peptide production using reporter strain. The red arrows denote the blue color appeared on an agar plate.

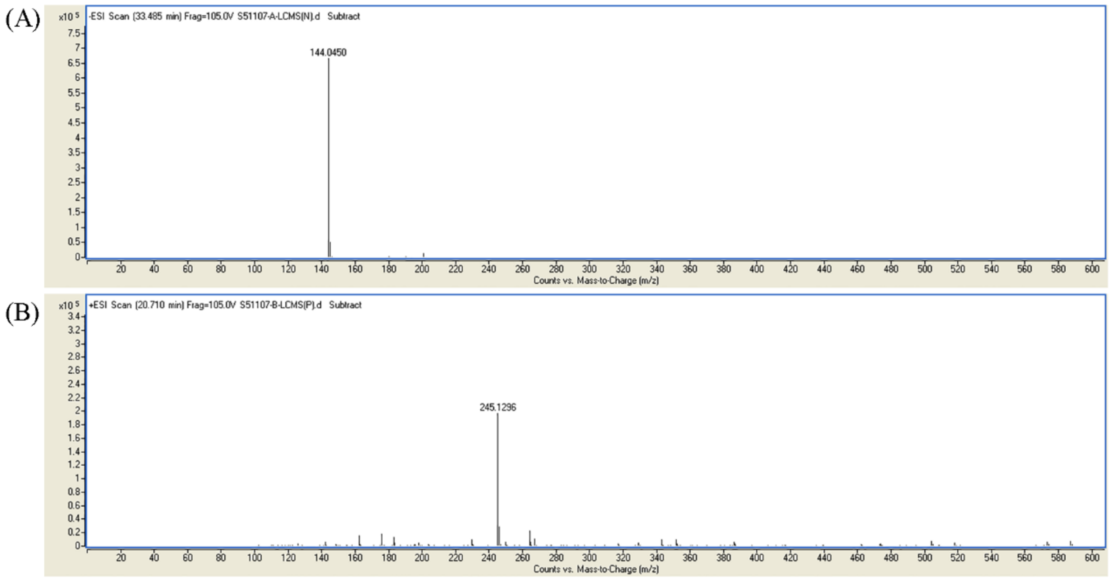

Supplementary Figure S8. HR-ESI-MS of algicidal compounds, S51107-A and S51107-B. S51107-A (A) and S51107-B (B) were operated in negative and positive mode with an Eclipse XDB-C18 column (Agilent Technologies HPLC 1290-MS 6230, USA), respectively.

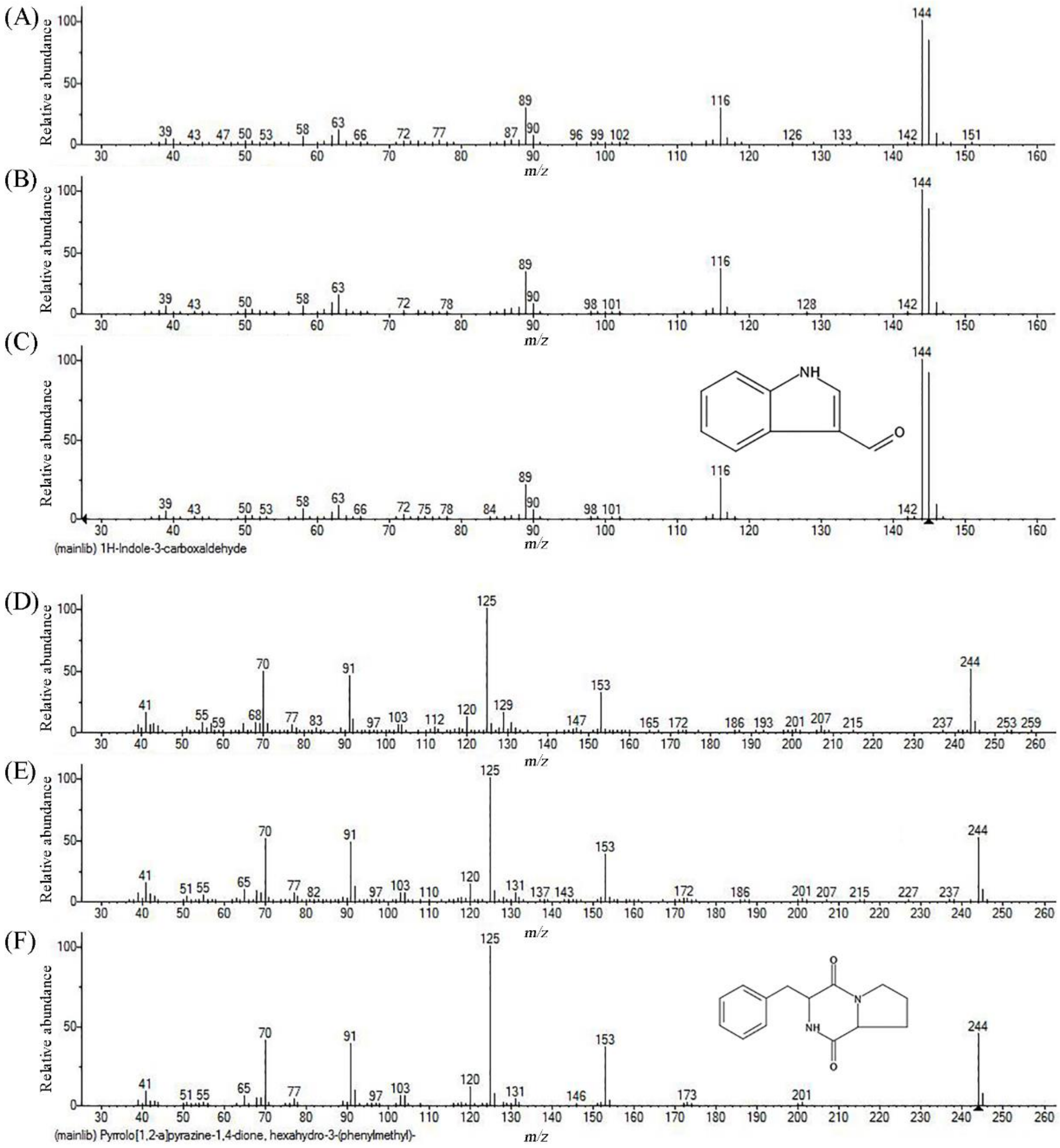

Supplementary Figure S9. EI gas mass spectra. EI gas mass spectra of algicidal compounds S51107-A (A), S51107-B (D), standard indole-3-carboxaldehyde (B), and standard 3-benzyl-hexahydro-pyrrolo[1,2-*a*]pyrazine-1,4-dione (E) by GC-MS analysis and the spectra of indole-3-carboxaldehyde (C) and 3-benzyl-hexahydro-pyrrolo[1,2-*a*]pyrazine-1,4-dione (F) in the GC-MS library. The chemical structures of indole-3-carboxaldehyde and 3-benzyl-hexahydro-pyrrolo[1,2-*a*]pyrazine-1,4-dione was revealed in the mass spectra from GC-MS library, respectively.

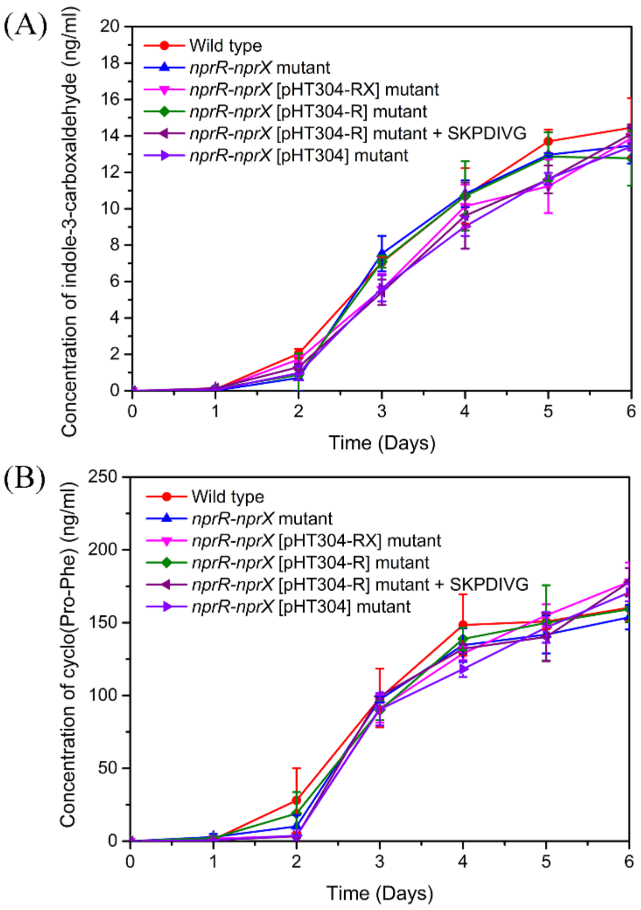

Supplementary Figure S10. Dynamics of the concentration of the algicidal compounds indole-3-carboxaldehyde (A) and cyclo(Pro-Phe) (B) during the algicidal process of *Bacillus* sp. strain S51107 and its mutants against *M. aeruginosa* 9110. Data are averages of three independent experiments (error bars are SD from mean values). Group A: wild type, *nprR-nprX* [pHT304-RX] mutant, *nprR-nprX* [pHT304-R] mutant + SKPDIVG; group B: *nprR-nprX* mutant, *nprR-nprX* [pHT304-R] mutant, *nprR-nprX* [pHT304] mutant.

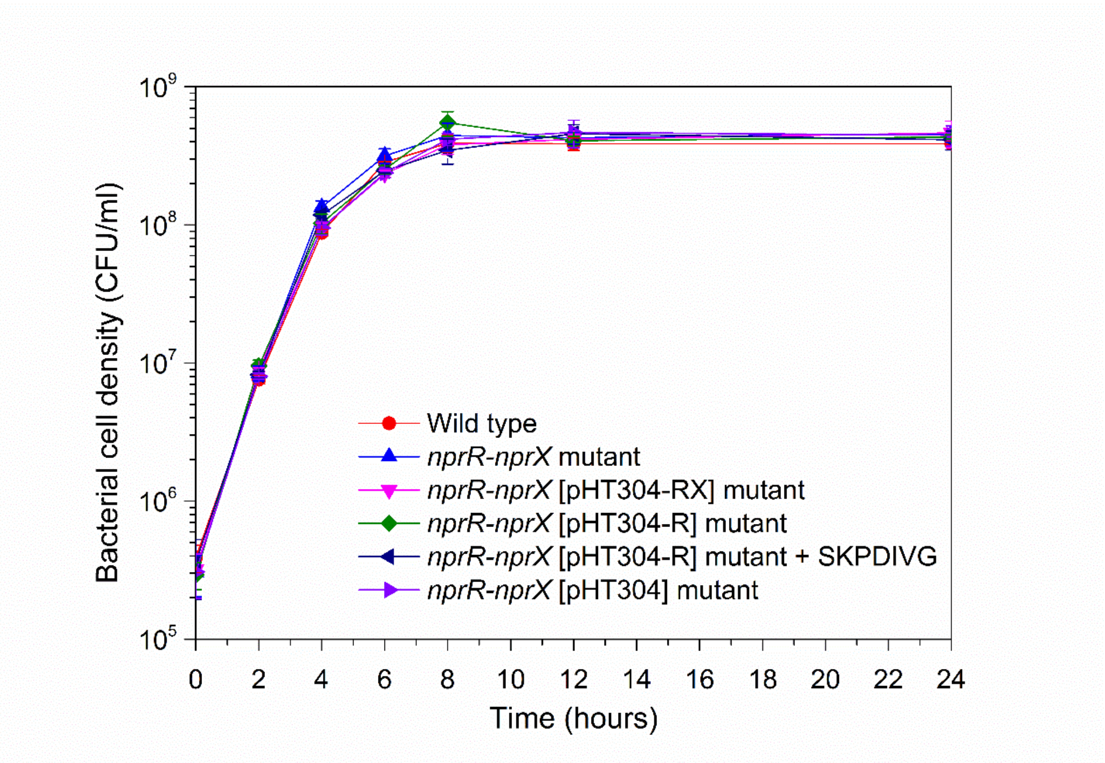

Supplementary Figure S11. Growth curves of *Bacillus* sp. strain S51107, *nprR-nprX* mutant, *nprR-nprX* [pHT304-RX] mutant, *nprR-nprX* [pHT304] mutant, *nprR-nprX* [pHT304-R] mutant and *nprR-nprX* [pHT304-R] mutant with 5 µM SKPDIVG. The cultures were grown at 30°C and 200 rpm in BEP medium. Data are averages of three independent experiments (error bars are SD from mean values).

SUPPLEMENTARY TABLES

Supplementary Table S1. Bacterial strains used in this study.

Strains	Relevant features[a]	Reference
Bacillus sp. strain S51107	Wild type algicidal strain isolated from Lake Taihu, China	This study
Bacillus sp. strain *nprR-nprX* mutant	*Bacillus* sp. strain S51107 mutant obtained by deletion of *nprR* and *nprX* genes	This study
Bacillus sp. strain *nprR-nprX* [pHT304-R] mutant	*nprX*-deficient strain, *Bacillus* sp. strain *nprR-nprX* mutant introducing the pHT304-R plasmid	This study
Bacillus sp. strain *nprR-nprX* [pHT304-RX] mutant	*Bacillus* sp. strain *nprR-nprX* mutant introducing the pHT304-RX plasmid	This study
Bacillus sp. strain *nprR-nprX* [pHT304] mutant	*Bacillus* sp. strain *nprR-nprX* mutant introducing the pHT304 plasmid	This study
Escherichia coli DH5α	F^- *Φ*80d*lacZ*ΔM15 Δ(*lacZYA-argF*)U169 *deoR recA1 endA1 hsdR*17($r_k^- m_k^+$) *phoA supE44* λ^- *thi-1 gyrA96 relA1*	(Hanahan, 1983)
Escherichia coli SCS110	*rpsL*(Str^r) *thr leu endA thi-1 lacY galK galT ara tonA tsx dam dcm supE44 Δ(lac-proAB)* [F′ *traD36 proAB lacI*^{q}*ZΔM15*]	(Jerpseth and Kretz, 1993)
Bacillus thuringiensis 407 Cry^- (*nprA'Z* ΔRX [pHT304-R])	Reporter strain, Tc^R, donated by Dr. Didier Lereclus from Institut Pasteur, Paris	(Perchat et al., 2011)

[a] Tc^R: tetracycline resistance.

Supplementary Table S2. Primers used in this study.

Primer name	Sequence	Restriction site[a]
27F	AGAGTTTGATCATGGCTCAG	/
1492R	GGTTACCTTGTTACFACTT	/
Npr1-F	GGCTTAAATCGACAGGATACTCTTCGG	/
Npr2-R	AAAATCGTTGCTCCGCGCGT	/
NPRX1-F	CGGGATCCGTAGAGGAGAAACCACC	BamHI
NPRX2-R	GGGTGGAGATTATAACATTGTCCTCCTCCATTCTC	/
NPRX3-F	TGGAGGAGGACAATGTTATAATCTCCACCCTTTCC	/
NPRX4-R	CATGCCATGGCAACTGTTCCAGACAATAC	NcoI
Npr1E-F	CCGGAATTCGTAGCCGTTCTCAAATGGAAGC	EcoRI
Npr2B-R	CGCGGATCCATGTTTATTCCTCCTTATTATCATTCATT	BamHI
Npr3B-R	CGCGGATCCGGAGATTATAAATTAACTTGTTCAACAG	BamHI
NprR-2	GCTAACACTAACGCTAAAC	/

[a] The restriction sites and the sticky ends are underlined.

Supplementary Table S3. Plasmids used in this study.

Plasmids	Relevant features[a]	Reference
pMAD	A thermosensitive shuttle plasmid in the construction of deletion mutants, which contains a temperature-sensitive origin of replication, Em^R, donated by Dr. Michel Debarbouille (Institut Pasteur, Paris) and Dr. Di Qu (Shanghai Medical College of Fudan University, China)	(Arnaud et al., 2004)
pHT304	Plasmid for gene complementation, *E. coli* and *B. thuringiensis* shuttle vector, Amp^R, Em^R, donated by Dr. Didier Lereclus (Institut Pasteur, Paris)	(Arantes and Lereclus, 1991)
pMAD-RX	Flanking regions of *nprR-nprX* genes cloned between the BamHI and NcoI restriction sites of the pMAD plasmid to generate internal deletion in the *nprR-nprX* genes	This study
pHT304-R	The wild type *nprR* gene with its endogenous promoters was amplified using Npr1E-F/Npr2B-R primers and strain S51107 chromosomal DNA as the template, and inserted between the EcoRI and BamHI sites of pHT304, Em^R	This study
pHT304-RX	The wild type *nprR-nprX* genes with their endogenous promoters were amplified using Npr1E-F/Npr3B-R primers and strain S51107 chromosomal DNA as the template, and inserted between the EcoRI and BamHI sites of pHT304, Em^R	This study

[a] Amp^R: ampicillin resistance; Em^R: erythromycin resistance.

Supplementary Table S4. ^{1}H and ^{13}C NMR data of algicidal compounds A and B at 600 and 151 MHz, respectively, in MeOD.

	A[a]			B[b]	
position	δ_C	δ_H (int., mult., *J* in Hz)	position	δ_C	δ_H (int., mult., *J* in Hz)
2	139.71	8.10 (1H, s)	1	166.90	
3	120.13		3	45.95	3.54 (1H, dt, 12.0, 8.4)<br>3.40-3.35 (1H, m)
3a	125.72		4	22.76	1.84-1.77 (2H, m)
4	123.61	8.16 (1H, d, 7.8)	5	29.39	2.12-2.06 (1H, m)<br>1.25-1.17 (1H, m)
5	122.40	7.24 (1H, td, 7.6, 1.0)	6	60.07	4.07 (1H, dd, 10.8, 6.4)
6	125.00	7.30–7.26 (1H, m)	7	170.90	
7	113.13	7.48 (1H, d, 8.0)	8		4.62 (1H, br s)
7a	138.94		9	57.70	4.45 (1H, t, 5.0)
8	187.42	9.89 (1H, s)	10	38.22	3.17 (2H, qd, 14.2, 5.0)
NH		4.59 (s)	Ar		
			1'	137.32	7.30-7.21 (5H, m)
			2', 6'	131.06	
			3', 5'	129.45	
			4'	128.08	

[a] The NMR data were consistent with reference (Amir-Heidari et al., 2007; Zhang et al., 2016).
[b] The NMR data were consistent with reference (Adamczeski et al., 1995; Wang et al., 2010; Chen et al., 2012).

REFERENCES

Adamczeski, M., Reed, A.R., and Crews, P. (1995). New and known diketopiperazines from the Caribbean sponge, *Calyx* cf. *podatypa*. *J. Nat. Prod.* 58(2), 201-208. doi: 10.1021/np50116a007.

Amir-Heidari, B., Thirlway, J., and Micklefield, J. (2007). Stereochemical course of tryptophan dehydrogenation during biosynthesis of the calcium-dependent lipopeptide antibiotics. *Organic Letters* 9(8), 1513-1516. doi: 10.1021/ol0701619.

Arantes, O., and Lereclus, D. (1991). Construction of cloning vectors for *Bacillus thuringiensis*. *Gene* 108(1), 115-119. doi: 10.1016/0378-1119(91)90495-W.

Arnaud, M., Chastanet, A., and Débarbouillé, M. (2004). New vector for efficient allelic replacement in naturally nontransformable, low-GC-content, gram-positive bacteria. *Appl. Environ. Microbiol.* 70(11), 6887-6891. doi: 10.1128/aem.70.11.6887-6891.2004.

Chen, J.H., Lan, X.P., Liu, Y., and Jia, A.Q. (2012). The effects of diketopiperazines from *Callyspongia* sp. on release of cytokines and chemokines in cultured J774A.1 macrophages. *Bioorg. Med. Chem. Lett.* 22(9), 3177-3180. doi: 10.1016/j.bmcl.2012.03.045.

Guo, X., Liu, X., Wu, L., Pan, J., and Yang, H. (2016). The algicidal activity of *Aeromonas* sp. strain GLY-2107 against bloom-forming *Microcystis aeruginosa* is regulated by *N*-acyl homoserine lactone-mediated quorum sensing. *Environ. Microbiol.* 18(11), 3867-3883. doi: 10.1111/1462-2920.13346.

Hanahan, D. (1983). Studies on transformation of *Escherichia coli* with plasmids. *J. Mol. Biol.* 166(4), 557-580. doi: 10.1016/S0022-2836(83)80284-8.

Jerpseth, B., and Kretz, B.L. (1993). SCS110: *dam*$^-$, *dcm*$^-$, *endA*$^-$ Epicurian coli® competent cells. *Strategies* 6(22), 15.

Li, Z., Lin, S., Liu, X., Tan, J., Pan, J., and Yang, H. (2014). A freshwater bacterial strain, *Shewanella* sp. Lzh-2, isolated from Lake Taihu and its two algicidal active substances, hexahydropyrrolo[1,2-a]pyrazine-1,4-dione and 2, 3-indolinedione. *Appl. Microbiol. Biotechnol.* 98(10), 4737-4748. doi: 10.1007/s00253-014-5602-1.

Lin, S., Pan, J., Li, Z., Liu, X., Tan, J., and Yang, H. (2014). Characterization of an algicidal bacterium *Brevundimonas* J4 and chemical defense of *Synechococcus* sp. BN60 against bacterium J4. *Harmful Algae* 37, 1-7. doi: 10.1016/j.hal.2014.05.002.

Perchat, S., Dubois, T., Zouhir, S., Gominet, M., Poncet, S., Lemy, C., et al. (2011). A cell-cell communication system regulates protease production during sporulation in bacteria of the *Bacillus cereus* group. *Mol. Microbiol.* 82(3), 619-633. doi: 10.1111/j.1365-2958.2011.07839.x.

Tian, C., Liu, X., Tan, J., Lin, S., Li, D., and Yang, H. (2012). Isolation, identification and characterization of an algicidal bacterium from Lake Taihu and preliminary studies on its algicidal compounds. *J. Environ. Sci. (China)* 24(10), 1823-1831. doi: 10.1016/S1001-0742(11)60983-2.

Wang, G., Dai, S., Chen, M., Wu, H., Xie, L., Luo, X., et al. (2010). Two diketopiperazine cyclo(PRO-PHE) isomers from marine bacteria *Bacillus subtilis* sp. 13-2. *Chemistry of Natural Compounds* 46(4), 583-585. doi: 10.1007/s10600-010-9680-8.

Yi, Y.-L., Lei, Y., Yin, Y.-B., Zhang, H.-Y., and Wang, G.-X. (2012). The antialgal activity of 40 medicinal plants against *Microcystis aeruginosa*. *J. Appl. Phycol.* 24(4), 847-856. doi: 10.1007/s10811-011-9703-2.

Zhang, C., Yi, Y.L., Hao, K., Liu, G.L., and Wang, G.X. (2013). Algicidal activity of *Salvia miltiorrhiza* Bung on *Microcystis aeruginosa*-Towards identification of algicidal substance and determination of inhibition mechanism. *Chemosphere* 93(6), 997-1004. doi: 10.1016/j.chemosphere.2013.05.068.

Zhang, L., Tu, Z.-c., Yuan, T., Wang, H., Xie, X., and Fu, Z.-f. (2016). Antioxidants and α-glucosidase inhibitors from Ipomoea batatas leaves identified by bioassay-guided approach and structure-activity relationships. *Food Chem.* 208, 61-67. doi: 10.1016/j.foodchem.2016.03.079.
